# Diversity and inclusivity in Australian dementia prevention research: A mixed methods review

**DOI:** 10.1002/trc2.70296

**Published:** 2026-07-18

**Authors:** Olivia E. Giameos, Maddison L. Mellow, Diana Karamacoska, Aaron Davis, Paul A Gardiner, Eddy Roccati, Kaarin J. Anstey, Nicolas Cherbuin, Jessica M. Adsett, Nikki‐Anne Wilson, Kylie Radford, Louise Lavrencic, Perminder S. Sachdev, Simone Reppermund, Suraj Samtani, Annabel P. Matison, Zara A. Page, Philip J. Batterham, Sophie C. Andrews, Alex Bahar‐Fuchs, Michelle K. Lupton, Andrew Pipingas, Adrienne Withall, Belinda M. Brown, Helen Macpherson, Hannah A. D. Keage, Britt Burton, James C. Vickers, David W Dunstan, Ashleigh E. Smith

**Affiliations:** ^1^ Alliance for Research in Exercise, Nutrition and Activity (ARENA) Research Centre, Allied Health and Human Performance Adelaide University Adelaide Australia; ^2^ NICM Health Research Institute Western Sydney University Westmead Australia; ^3^ School of Architecture and Built Environment College of Creative Arts Design and Humanities Adelaide University Adelaide Australia; ^4^ The Florey Institute of Neuroscience and Mental Health Heidelberg Australia; ^5^ School of Public Health The University of Queensland University Drive Brisbane Australia; ^6^ Wicking Dementia Research and Education Centre University of Tasmania Hobart Australia; ^7^ UNSW Ageing Futures Institute University of New South Wales Sydney Australia; ^8^ School of Psychology Faculty of Science University of New South Wales Sydney Australia; ^9^ Neuroscience Research Australia (NeuRA) Sydney Australia; ^10^ National Centre for Epidemiology and Population Health Australian National University Canberra Australia; ^11^ QIMR Berghofer Medical Research Institute Brisbane Australia; ^12^ Centre for Healthy Brain Ageing (CHeBA), Discipline of Psychiatry and Mental Health, School of Clinical Medicine Faculty of Medicine and Health UNSW Sydney Sydney Australia; ^13^ Neuropsychiatric Institute The Prince of Wales Hospital Sydney Australia; ^14^ Centre for Mental Health Research The Australian National University Canberra Australia; ^15^ Thompson Institute University of the Sunshine Coast Birtinya Australia; ^16^ School of Psychology Deakin University Melbourne Australia; ^17^ Department of Psychiatry University of Melbourne Melbourne Australia; ^18^ Centre for Mental Health and Brain Sciences Swinburne University of Technology Hawthorn Australia; ^19^ Centre for Healthy Ageing Health Futures Institute Murdoch University Murdoch Australia; ^20^ Centre for Precision Health Edith Cowan University Joondalup Australia; ^21^ Institute for Physical Activity and Nutrition School of Exercise and Nutrition Sciences Deakin University Burwood Australia; ^22^ School of Psychology College of Education Behavioural and Social Sciences Adelaide University Magill Australia; ^23^ Physical Activity Laboratory Baker Heart and Diabetes Institute Melbourne Australia

**Keywords:** ageing, dementia, diversity, minoritized populations, prevention

## Abstract

Minoritized communities are disproportionately impacted by modifiable dementia risk factors but are underrepresented in dementia research globally. In Australia, the number of dementia prevention cohort and intervention studies has rapidly increased in the past two decades, yet the representation of minoritized groups has not been synthesized. The aims of this mixed‐methods review were to summarize the demographic characteristics of participants involved in Australian dementia prevention‐focused cohort and intervention studies. A systematic search of published literature, funded grant outcomes, and clinical trial registrations was conducted in October 2023 and updated in February 2025 to identify any Australia‐based cohort or intervention studies which focused on dementia prevention. Data custodians of eligible studies were contacted and invited to submit de‐identified participant‐level data for these studies. The demographic characteristics of participants involved in published manuscripts and submitted participant‐level data were synthesized. These data were then presented to 23 Australian dementia prevention researchers during a 2‐h workshop in March 2025. Using co‐creation methodologies, the workshop aimed to generate consensus‐based recommendations for governments, institutions, and individual researchers to improve inclusivity and representativeness in future dementia prevention research. Twenty‐eight published dementia prevention studies were summarized alongside fourteen studies for which de‐identified participant‐level data were submitted. Compared to the modern Australian population, participants enrolled in dementia prevention studies have been predominantly female, highly educated, and less diverse in terms of culture, language, ethnicity and gender. Thirty‐seven recommendations were generated by Australian researchers and ranked by importance, providing actionable changes for government and institutional policy and researcher practice. This review identified that participants enrolled in Australian dementia prevention research studies do not accurately represent those most at risk for dementia in the general population. Changes to recruitment and engagement practices and policies are recommended to improve the representativeness and inclusivity of Australian dementia prevention research.

## Highlights

- Australian dementia prevention research cohorts don't accurately reflect general population.
- Generalizability of evidence based on existing cohorts may be limited.
- Thirty‐seven recommendations to improve diversity and representation were generated by experts.
- Recommendations span individual researcher, institutional and government levels.

## 1 BACKGROUND

Without significant advancements in treatment or preventive strategies, the number of people living with dementia worldwide is estimated to exceed 150 million by 2050.^1^ Promisingly, 14 potentially modifiable health and lifestyle risk factors have been identified which may prevent or delay up to 45% of dementia cases globally, and these factors are frequently targeted in dementia prevention efforts.^2^ However, it is increasingly recognized that risk factor modifiability is not uniform, and opportunities for dementia prevention are not “one‐size‐fits‐all.” Environmental, systemic, and policy‐level barriers to accessing and engaging with dementia prevention efforts have been identified, including racial, cultural, and socio‐economic disparities in access to science (e.g., clinical trials) and healthcare,^3^, ^4^ as well as other social and political determinants of dementia including the food environment, housing and sanitation, pollution, the built and natural environments, social inclusion, intergenerational disadvantage, income, and employment.^5^ Relatedly, many modifiable dementia risk factors disproportionately impact people from low and middle‐income countries, Indigenous peoples, culturally and linguistically diverse (CALD) communities, people living in regional/rural/remote areas, and other underserved populations.^2^ Herein, we use the term “minoritized” rather than “minority” to reflect that, for such communities, their underrepresentation in research and overrepresentation in adverse health outcomes stems from systemic and social inequality, rather than their size or characteristics.^6^

There are several key examples of such disparities in Australia. Recent modelling showed differences by population groups in Australia attributed to 11 modifiable risk factors: less education, obesity, hearing loss, hypertension, excessive alcohol, physical inactivity, type 2 diabetes, daily smoking, depression, air pollution, and social isolation. This highlighted substantial opportunity for prevention, particularly for Aboriginal and Torres Strait Islander peoples for whom this set of factors accounted for nearly 45% of dementia cases (compared to 38.2% estimated for the total Australian population).^7^ Developing targeted prevention initiatives for this population is a key health priority, given that dementia prevalence rates in Aboriginal and Torres Strait Islander communities are estimated to be three to five times higher than non‐indigenous Australians.^8^, ^9^, ^10^ Australia is a CALD nation, with approximately one‐third of the population born overseas.^11^ Sue See et al.^7^ estimated 36.4% of dementia cases among Australians with European ancestry and 33.6% of dementia cases among Australians with Asian ancestry were attributable to the same 11 modifiable risk factors. Of note, the population‐attributable fraction (PAF) estimates across individual risk factors were varied: for example, the PAF for type 2 diabetes ranked higher, but depression and obesity ranked lower, for Australians with Asian ancestry compared to those with European ancestry.^7^ Similarly, PAFs were higher for smoking, diabetes, and less formal education for Aboriginal and Torres Strait Islander peoples versus other groups.^6^

Recent cross‐sectional time‐series analyses by Welberry et al.^12^ demonstrated that irrespective of age, sex, and year of survey, Australians in the lowest income group had higher prevalence of almost all modifiable dementia risk factors (except for alcohol consumption), and subsequently, a greater combined PAF (52.2%) compared to Australians in the highest income group (PAF: 43.4%). As a result, to achieve equivalent risk reduction, intervention strategies must consider differences in culture, language, social inclusion, socio‐economic status, and living conditions, while targeting the most relevant underlying risk factors for that population.^7^ Finally, although the contribution of modifiable risk factors to dementia prevalence in other priority population groups such as people with disability, rural/regional/remote residents, and lesbian, gay, bisexual, transgender, and intersex (LGBTI+) communities has not been modelled specifically in Australia, previous evidence suggests that these populations are at higher risk of chronic conditions, cognitive decline, and face unique barriers to healthcare access,^13^, ^14^, ^15^, ^16^ which collectively may place them at greater risk for dementia. Indeed, evidence from the United States demonstrated that transgender and non‐binary older adults have greater overall late‐life dementia risk due to modifiable factors compared to cisgender men and women.^17^

Despite Australia's increasing diversity and clear trends of the disproportionate prevalence of dementia and its risk factors among minoritized communities, these populations remain under‐represented in dementia research.^8^, ^18^, ^19^ Most evidence to date on a global scale has come from high‐income, urban, well‐educated, or non‐marginalized populations.^2^, ^3^, ^5^, ^7^, ^20^ Consequently, prevention strategies and public health messaging arising from current cohort and intervention studies may be limited in their generalizability and effectiveness for diverse ageing populations. This is a long‐standing and well‐recognized issue, with several call‐to‐action papers providing clear recommendations for the recruitment, inclusion and retention of underrepresented populations in dementia research previously.^20^, ^21^, ^22^ The number of dementia prevention cohort and intervention studies in Australia has grown substantially in the past two decades. Thus, characterizing the demographic profiles of samples on which the existing evidence is based is a key priority to facilitate clear guidelines for future study planning (e.g., recruitment, sub‐group analyses, and inclusion criteria), at a time when dementia prevention is a local and global health priority.

The aims of this mixed‐methods review were two‐fold. First, we aimed to summarize the demographic characteristics (and demographic reporting practices) of participants involved in Australian dementia prevention research, using both published cohort‐level data and participant‐level data from Australian longitudinal cohort and intervention studies. Then, using a vision workshop (co‐creation methodology), we aimed to generate and prioritize recommendations through consensus among Australian dementia prevention researchers, that could inform future recruitment and reporting to improve representativeness and diversity of participants in Australian dementia prevention studies.

## 2 METHODS

### 2.1 Ethics

This mixed‐methods review was conducted in accordance with the Preferred Reporting Items for Systematic Reviews and Meta‐Analyses (PRISMA) statement, where applicable (Supplementary File 1). The project was prospectively registered on Open Science Framework (Registration ID: https://doi.org.10.17605/OSF.IO/4QBJ5). Ethics approval was sought for the component of the study involving contacting the researchers identified in the literature and grant search to request and synthesize de‐identified participant‐level demographic data. Ethics approval was granted by the University of South Australia Human Research Ethics Committee (Approval ID 206076). Ethics approval was not sought for the vision workshop as this was conducted with manuscript co‐authors.

To enable the transfer and analysis of de‐identified participant‐level data, the data custodian (or the institution submitting the data) established data sharing agreements where required. These agreements were developed in line with each custodian's institutional guidelines and ethical obligations (e.g., seeking approval from the Aboriginal Health and Medical Research Council). Agreements included specific project details (including variables), the researchers who were approved to view and analyze the submitted data, and any necessary acknowledgements of the study, and were signed by both parties prior to the transfer of any data.

### 2.2 Inclusion criteria

Studies were included if they met the following criteria: the sample was recruited from and research was conducted in Australia; the study explicitly stated in the manuscript or grant summary that it was focused on dementia prevention/risk reduction and/or brain or cognitive health, and targeted (or studied) one or more modifiable dementia risk factors as outlined by the Lancet Commission's 2020 dementia prevention, intervention, and care report^23^ (education, hearing loss, traumatic brain injury, hypertension, alcohol consumption, obesity, smoking, depression, social isolation, physical inactivity, air pollution, or type 2 diabetes; 2020 iteration used due to timing of data collection and synthesis); the research design was a longitudinal cohort study or intervention (e.g., randomized controlled trial [RCT] or single group pre–post intervention); and recruitment of participants was completed. Due to the focus on prevention, studies had to include participants without dementia but were still included if a sub‐group of participants were living with dementia. Large cohort studies or hospital records with a generalized health focus were excluded, as well as cross‐sectional observational studies, participant registries (i.e., with ongoing recruitment), pharmaceutical studies (e.g., drug or supplement trials), systematic reviews and meta‐analyses, conference abstracts, letters to editors, and dementia risk screening tool validation studies.

### 2.3 Data acquisition strategy

#### 2.3.1 Published manuscripts and clinical trial registries

A multi‐stage search and screening process was conducted to identify relevant Australia‐based longitudinal cohort and intervention studies focused on dementia prevention. First, a search of the online databases OVID (Medline, EMBASE, PsycInfo) and EBSCO (CINAHL) was conducted on October 31, 2023, using the search strategy outlined in Supplementary File 2. Using the same search strategy, a follow up search was conducted on February 11, 2025, to capture all manuscripts published after the initial search date. To supplement the initial literature search, clinical trial registries (Clinicaltrials.gov, ANZCTR, and CENTRAL) were searched on November 13, 2023, to identify other potentially relevant Australia‐based dementia prevention cohort and interventions studies, and where possible, published manuscripts related to clinical trial registrations were located. The manuscripts identified from all searches were exported to EndNote where duplicates were removed. Manuscripts were then imported to Covidence where another duplicate removal was completed before screening. Title and abstract and full‐text screening stages were completed by two independent researchers (O.E.G. and M.L.M.). Any conflicts were resolved through discussion between the researchers with assistance from an additional researcher where conflicts could not be resolved (A.E.S.).

To avoid duplication of cohort and intervention studies in the published data analysis (i.e., multiple manuscripts using the same dataset), we used a hierarchical method to select manuscripts from which demographic characteristics would be synthesized, with a preference for published cohort profiles. Where cohort profiles were not available, the published manuscripts which captured the greatest number of participants from the original cohort or intervention study were included in the data synthesis.

#### 2.3.2 Funded dementia prevention‐focused grants

To extend the initial database searches, an additional search of the published outcomes of funding rounds and grants related to dementia prevention in Australia was conducted (October 2023). Specifically, the funding bodies searched included the Dementia Centre for Research Collaboration, the National Health and Medical Research Council (including the Boosting Dementia Research Initiative), the Medical Research Future Fund, and the Dementia Australia Research Foundation. All relevant research project titles, chief investigator names, contact emails, and the brief research explanation were collated from the funding announcements in an Excel spreadsheet. Two independent researchers screened these grants for eligibility for inclusion (O.E.G. and M.L.M.). Conflicts were discussed with a senior researcher (A.E.S.) if they could not be resolved.

#### 2.3.3 Follow‐up surveys to obtain participant‐level data

Recognizing that many studies may collect more variables than they publish, we also contacted data custodians for access to any de‐identified participant‐level demographic data they were able to share, for inclusion in a supplementary analysis. This included those for whom sample‐level data were already available (i.e., studies that were already captured in the published data analysis previously described). Data for identified cohort and intervention studies were requested via a Research Electronic Data Capture (REDCap) survey.^24^ All corresponding authors of relevant manuscripts identified in the systematic literature search, and lead project investigators from the funded grant search, were emailed with this request. The de‐identified participant‐level data requested included: age, gender, sex, education level, sexual orientation, nationality, ethnicity, Aboriginal and Torres Strait Islander status, socio‐economic status, geographic location, relationship status, first language, preferred language, country of birth, methods of recruitment and data analysis, research team characteristics, and funding source(s). Respondents were prompted to ensure their ethics approval allowed sharing of de‐identified participant‐level data prior to submission.

### 2.4 Data extraction and synthesis of published cohort‐level demographic data

Data were extracted from published manuscripts by one researcher (O.E.G.) using REDCap software and checked for accuracy by another (M.L.M./A.E.S.). For continuous variables (e.g., age, years of education), means and standard deviations (or median and inter‐quartile range for skewed data) were extracted. For categorical variables, frequency and proportion were extracted. Data at the sample level were extracted for the following variables (where available): age; sex; gender; education level; sexual orientation; ethnicity; Aboriginal and Torres Strait Islander status; socio‐economic status (including annual income, employment status, Socio‐Economic Indexes For Areas (SEIFA), Index of Relative Socioeconomic Advantage and Disadvantage (IRSAD), Australian Bureau of Statistics (ABS) skill level of job, living circumstances, or home duties); relationship status; geographical location (including a specific state/city and their remoteness as per the Modified Monash Model classification^25^); first language; preferred language; and country of birth. The geographical location of the participants was only recorded if it was explicitly reported in the manuscript, such that even if the research was conducted by a specific institution for which the location was known, it was not assumed that participants were recruited locally. Remoteness was classified based on the Modified Monash Model^25^ whenever the specific recruitment location (e.g., postcode or suburb where participants resided) was provided; otherwise, the remoteness classifications stated (e.g., “rural” or “metropolitan”) were retained.

Further information was extracted to provide context to the demographic representation of the sample, including recruitment method (e.g., purposive vs. random sampling), data analysis approach, research team characteristics (e.g., sex, ethnicity, profession; only if reported), years of data collection, and year that the manuscript was published. For intervention studies, variables which were controlled for in the analyses (e.g., age and sex as covariates) were also extracted. For larger cohort profile papers where statistical analyses were not reported, the empirical papers arising from this cohort (frequently reported in the cohort profile manuscript) were used to identify which demographic variables had been included in statistical analyses. The data extraction was focused on reported variables; therefore, the extraction included only the variables for which data were provided in the publication, regardless of whether the research team reported that the variable was collected as part of the larger study.

Data synthesis was completed using R software (version 4.5.1).^26^ All data were descriptively synthesized, and no inferential statistical analyses were conducted. For the published cohort‐level data, we summarized the total number of published studies that had reported each demographic variable in their manuscripts and generated descriptive summaries of reported demographic variables.

### 2.5 Synthesis of participant‐level demographic characteristics

To facilitate synthesis of the submitted de‐identified participant‐level demographic data, some variables were harmonized across studies. A comprehensive summary of how participant‐level data were received and manipulated for synthesis is provided in Supplementary File 3. Briefly, we re‐classified highest level of education/highest qualification data into the following categories: (1) high school or lower; (2) certificate/associate degree/diploma/trade certificate; (3) Bachelor's degree; (4) higher University degree (Masters, post‐graduate diploma, or postgraduate degree); or (5) other/don't know. Relationship status was classified into the following categories: (1) partnered (married, living with a partner, or de‐facto); (2) divorced/separated; (3) widowed; (4) never married/single; or (5) other/don't know. All other variables (age, gender, sex, total years of education, preferred language, primary/first language, country of birth, ethnicity, Aboriginal and Torres Strait Islander status, socio‐economic status, and sexual orientation) remained in their original format for synthesis.

For both published cohort‐level data and de‐identified participant level data, the terms “female” and “male” were used to refer to the sex of a participant, and “man,” “woman,” “non‐binary,” and “transgender” were used to represent gender. It should be noted that studies used conflicting terminology when reporting these variables. For clarity, where studies explicitly reported that “sex” and/or “gender” were collected, the data were classified accordingly, regardless of whether the variables used the terminology described above. For studies which did not explicitly report which variable they collected, the terminology used in their reporting (female and male vs. woman and man) guided the classification.

### 2.6 Data interpretation and development of recommendations

Following data synthesis, a 2‐hour vision workshop was held online (via video conferencing) with 23 Australian dementia prevention researchers, all of whom were invited to participate as co‐authors on the manuscript (March 25, 2025). Researchers (*n* = 33 initially contacted) were invited if they submitted de‐identified participant‐level data, or if they indicated interest in participating in the research project on the data request form. Workshop attendees represented 13 Australian research institutions across six out of the eight Australian states and territories (South Australia, New South Wales, Victoria, Queensland, Tasmania, and Australian Capital Territory). The workshop presented the results from the published data synthesis, then used a strategic foresight approach to collaboratively explore the potential implications of the findings.^27^ Visioning activities (described below) were used to explore both projected and possible futures for dementia prevention research,^28^ and to develop and prioritize recommendations for collection and reporting of demographic data in future research.

The workshop was co‐facilitated by A.D. (a researcher with extensive co‐design experience) and O.E.G., with breakout group discussions led by O.E.G., M.L.M., D.K., and A.E.S. The individual workshop activities were developed by A.D. and O.E.G. using Miro.^29^ To reduce the bias associated with facilitator scribing, the data collected during the workshop used democratized participant‐led documentation to empower each participant to contribute to key future priorities. Participants were guided through four tasks that led to the formation of recommendations.

Task 1 involved responding to the following prompts: “Why do we collect the data we are?”; “Why are we publishing the data we are?”; “Why don't we collect other types of demographic data?”; and “Why don't we publish other types of demographic data?.” In Task 2, participants then considered the implications (both positive and negative) of collecting and publishing various types of demographic data through social, economic, political, legal and ethical lenses. Task 3 involved extrapolation of the potential long‐term positive and negative consequences of both maintaining current practices, or of improving demographic reporting practices and increasing cohort diversity and representativeness. Finally, in Task 4, participants used their discussions from the previous tasks to develop recommendations that could be implemented to improve current practices at three scales: by governments, by institutions, and by individual researchers. We note that the definition of “institution” was kept broad and non‐specific, but some examples were provided to workshop participants for guidance (e.g., institutions, societies, or organizations). Workshop materials are presented in Supplementary File 4.

Following the workshop, all Miro boards were saved, and data for each activity were downloaded into a password protected Excel worksheet. Data were analyzed using reflexive thematic analysis with coding underpinned by constructivist epistemology.^30^ Coding of themes was performed individually by O.E.G. and A.E.S. and verified by A.D. From this, a series of proposed recommendations were developed. These recommendations were presented to workshop participants in the form of a follow‐up online survey distributed using Qualtrics (Supplementary File 5). Participants were asked to rank recommendations by importance, and to review each recommendation against the highest (“whole‐heartedly endorse”) and lowest (“veto”) levels of an eight‐point scale, developed from Brause.^31^ A multiple criteria decision analysis was used to rank the recommendations by importance for presentation in the manuscript.^32^ Recommendations with the same ranking were further sorted by both whole‐hearted endorsement votes and by veto votes, and recommendations with >2 “veto” votes were removed from the list.

## 3 RESULTS

### 3.1 Cohort and intervention studies included in published data synthesis

The initial systematic literature search (2023) identified 351 published manuscripts for title and abstract screening, and the follow‐up search (2025) yielded 20 additional manuscripts for title and abstract screening (371 total). Of these, 126 progressed to full‐text screening. Concurrently, 258 funded Australian dementia prevention grants were screened, of which 72 were identified as relevant. In addition, 69 clinical trial registrations were screened, resulting in 13 identified as relevant. Following screening of grants and clinical trial registrations, seven additional studies were identified which were missed in the initial literature search. Following full‐text screening, 28 unique studies were included in published data analyses (see Supplementary File 6 for PRISMA flow diagram).

Among the 28 unique studies were 15 longitudinal cohort studies: Australian Longitudinal Study of Ageing (ALSA^33^); Sydney Memory and Ageing Study (MAS^34^); Personality and Total Health Through Life (PATH^35^); The Island Study Linking Ageing and Neurodegenerative Disease (ISLAND^36^); Women's Healthy Ageing Project (WHAP^37^); Australian Imaging Biomarkers and Lifestyle Flagship Study of Ageing (AIBL^38^); Melbourne Longitudinal Studies on Healthy Ageing (MELSHA^39^); ACTIVate^40^; Prospective Imaging Study of Ageing (PISA^41^); Tasmanian Healthy Brain Project (THBP^42^); Older Australian Twins Study (OATS^43^); Canberra Longitudinal Study (CLS^44^); The Sydney Centenarian Study (SCS^45^); Hunter Community Study^46^; and Koori Growing Old Well Study (KGOWS^47^). Additionally, 13 intervention studies were included: Lifestyle Intervention Study for Dementia Risk Reduction (LEISURE^48^); Body Brain Life (BBL^49^); BRAIN BOOTCAMP Research Program^50^; MedPork trial^51^; MedDairy trial^52^; Body Brain Life‐General Practice (BBL‐GP^53^); Body Brain Life for Cognitive Decline (BBL‐CD^54^); Promoting Healthy Ageing with Cognitive Exercise (PACE^55^); MedWalk^56^; Protein Omega‐3 and Vitamin D Exercise Research Study (PONDER^57^); Rainey‐Smith et al.^58^; Formica et al.^59^; and Danthiir et al.^60^

### 3.2 Published cohort‐level data reporting practices and participant demographics

The included studies were initiated between 1990 and 2022 and spanned 33,191 participants (sample size range: 33–7,485). A complete list of the included studies and their characteristics (study design, year of study initiation, year of affiliated publication, and sample size) is provided in Supplementary File 7.

**FIGURE 1:**
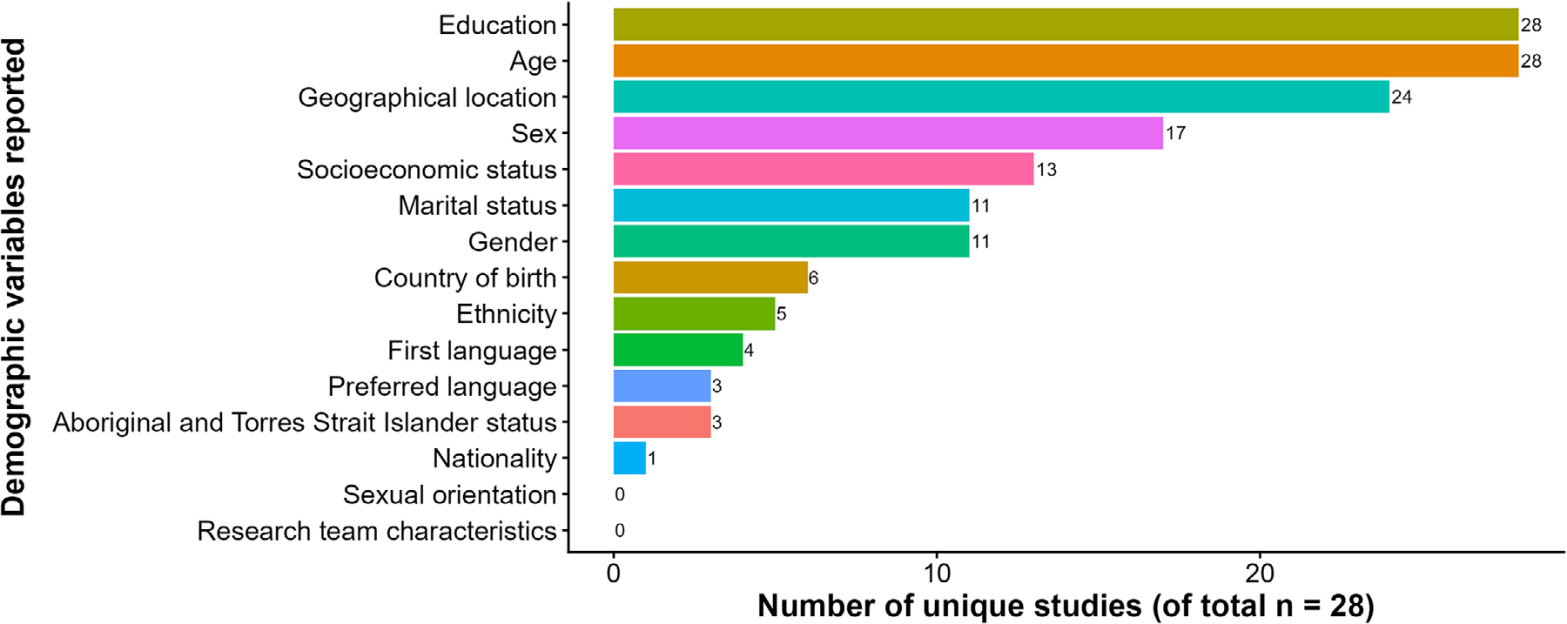
Summary of participant demographic characteristics reported by published dementia prevention cohort studies (*n* = 15) and intervention studies (*n* = 13).

In the published manuscripts, the most frequently reported demographic variables were age, education, and geographical location (Figure 1). Variables relating to the cultural and linguistic diversity of the sample were among the least frequently reported variables (i.e., Aboriginal and Torres Strait Islander status [10.7%], preferred language [10.7%], first language [14.3%], ethnicity [17.9%], and nationality [3.6%]). A more detailed overview of reported demographic variables can be viewed in Supplementary File 8.

Among the published cohort‐level datasets, the average age of participants was 68.0 ± 13.9 years (mean of means and medians). Education data were reported by 28 studies, with the most frequently reported variable being high school completion (reported by 16 studies). Fourteen studies (including 8,185 participants) reported the mean total years of education of their sample (14.0 ± 2.3 years; mean of means and medians). Across 24 studies (*n* = 23,616), 75.0% of participants had >12 years of education (*n* = 17,706), and 25% had <12 years of education (*n* = 5,910).

Twenty‐four studies reported the geographical location (Australian state/territory of recruitment) of their sample (27,804 participants). New South Wales was the most frequently reported site of recruitment (nine studies), followed by Victoria (six studies), South Australia (five studies), Australian Capital Territory (five studies), Queensland (two studies), Tasmania (two studies), and Western Australia (one study). No studies explicitly reported recruiting participants from the Northern Territory. Remoteness was reported explicitly by five studies and ascertained for 57.3% of participants (*n* = 19,005), of which 1.9% (*n* = 372) were from a regional, rural or remote location, and 98.1% from a major city (*n* = 18,633). Remoteness was not reported for 42.7% of participants (*n* = 14,186).

Seventeen studies reported participants’ sex (17,357 total participants). Within these studies, 61.4% were females (*n* = 10,662) and 38.6% were males (*n* = 6,695). Eleven studies reported the gender of their sample. Of the 15,834 total participants, 61.6% were women (*n* = 9.752), 38.5% were men (*n* = 6,092), and 0.03% of participants (all from one study) were classified as “other.” Whilst every study reported one of sex or gender, no studies reported both the sex and gender identity of participants.

Eleven studies reported the relationship status of their participants. Of these 18,198 participants, 64.4% were partnered (married/living with a partner/de‐facto; *n* = 11,726), 19.9% were separated/divorced (*n* = 3,629), 12.8% had never been married (*n* = 2,321), <1% were single (*n* = 67), and 1.8% were classified as “other” (*n* = 327).

The most frequently reported demographic characteristic relating to cultural and linguistic diversity was country of birth, reported by six studies (totaling 3,245 participants). Australia was the predominant country of birth (*n* = 1,747; 53.8%). Of the 2,203 participants (from four studies) for which first language was reported, 89.5% spoke English as their first language (*n* = 1,971). Similarly, of the 2,772 participants for which preferred language was reported, 95.4% reported English as their preferred language (*n* = 2,645; three studies). Among the three published studies which reported Aboriginal and Torres Strait Islander status (*n* = 4,572), the proportion of participants identifying as Aboriginal or Torres Strait Islander ranged between 1.9% and 100% (overall proportion across three studies = 9.0%).

Participant socio‐economic status was reported by 13 studies. The most frequently reported measures were employment status (including employed part time/full time, ever employed, unpaid work, unemployed, retired; *n* = seven studies) and living circumstances (including in community alone/with others, own home, retirement village, hostel villa, retirement home; *n* = seven studies), followed by indices of relative socio‐economic advantage or disadvantage (IRSD/IRSAD; *n* = two studies), home duties (*n* = two studies), annual income (*n* = two studies), and Australian Bureau of Statistics skill level (*n* = two studies).

Notably, participant nationality was only reported by one study, and sexual orientation was not reported in any studies. Additional demographic characteristics which are not reflected in the categories above included past prison/police custody which was reported by one study.

Twenty‐three studies reported including a demographic variable in their statistical analysis (e.g., as a predictor/covariate). The most common variables included in analyses were age (*n* = 19 studies), sex (*n* = 15 studies) and education (*n* = 13 studies), whilst gender (*n* = 8 studies), socio‐economic status (*n* = 3 studies), geographical location (*n* = 2 studies), ethnicity (*n* = 2 studies), country of birth (*n* = 1 study), and relationship status (*n* = 1 study) were less frequently accounted for in statistical analyses. Variables related to cultural and linguistic diversity of the sample (e.g., first language, Aboriginal and Torres Strait Islander status, and nationality) were not reported in any statistical analyses.

Recruitment method was reported by 25 studies. The most common methods of recruitment were print and radio advertisement (*n* = 15 studies), recruitment through the electoral roll (*n* = 7 studies), “random selection” (*n* = 5 studies), social media (*n* = 4 studies), and word‐of‐mouth (*n* = 4 studies). Seventeen studies used more than one method of recruitment.

### 3.3 Submitted participant‐level data: reporting practices and participant demographics

From the 98 authors contacted to request submission of de‐identified participant‐level data, 14 datasets were submitted, 10 of which were also captured in the published data analysis. The 14 participant‐level datasets included nine longitudinal cohort studies: The Island Study Linking Ageing and Neurodegenerative Disease (ISLAND^36^); The Canberra Longitudinal Study^44^; Prospective Imaging Study of Ageing: Genes, Brain and Behaviour (PISA^41^); ACTIVate^40^; The Older Australian Twins Study (OATS^43^); the Sydney Memory and Ageing Study (MAS^34^); the Sydney Centenarian Study (SCS^45^); Koori Growing Old Well Study (KGOWS^47^); and Personality and Total Health Through Life (PATH^35^); and five intervention studies, including Mind Your Nose,^61^ the Lifestyle Intervention Study for Dementia Risk Reduction (LEISURE),^48^ RESCUE,^62^ OPTIMISE Your Health,^63^ and MedWalk.^56^

Approximately 90 contacted authors did not submit participant‐level demographic data for a number of reasons, including: they had not yet produced a publication using the dataset; their ethics agreement did not permit data sharing; the data were too expensive to access; recruitment was incomplete; the grant was not directly related to dementia prevention/risk reduction; the resulting publication did not adhere to the inclusion criteria of the current study; or the data were no longer accessible. Fifty‐two authors provided no response.

The 14 studies (nine cohort and five intervention studies) included in participant‐level synthesis spanned 26,659 participants. Age (59.1 ± 15.8 years, range 20–106 years) and at least one measure of educational attainment was provided by all studies, followed by country of birth (*n* = 11 studies) and gender (*n* = 10 studies). A summary of the data (and variable structures) provided for participant‐level analysis is provided in Supplementary File 3.

A measure of either total years of education or highest education level/qualification was provided by all studies. Across eight studies (8,338 participants), the mean total years of education was 13.3 ± 5.0 years. There was considerable variability in the classifications used to measure highest education level/qualification (see Supplementary File 3 for classifications used). Following data harmonization, among 10 studies (23,848 participants) who provided these data, the majority (29.3%) of participants had a certificate, associate degree, diploma or trade, followed by high school or lower (26.3%), higher university degrees (masters, post‐graduate diploma, or PhD; 20.3%), bachelor's degree (19.4%), or “other” (4.7%).

Birth country data were available for 12,508 participants across 11 studies. Australia was the country of birth for 80.1% of participants (*n* = 10,022), followed by the United Kingdom (10.2%, *n* = 1,271), and New Zealand (1.4%, *n* = 176). South American (<1%), Asian (<1%), African (∼1%), and North American (∼1%) countries were the least represented in country of birth data.

Of the 23,822 participants for which gender data were available (*n* = 10 studies), 63.2% of participants (*n* = 15,050) were women (or female), and 36.8% (*n* = 8,772) were men (or male). Sex data were available from four studies (*n* = 2,589 participants), with 61.9% of participants identified as female (*n* = 1,604) and 38.0% as male (*n* = 985). No studies provided participant‐level data on both gender and sex.

Six studies (*n* = 10,859 participants) provided data on Aboriginal and Torres Strait Islander status. One study included only Aboriginal and Torres Strait Islander participants (*n =* 336^47^), three studies collected these data through a specific question about whether participants identify as Aboriginal or Torres Strait Islander, and two studies captured these data through a multiple‐choice option available within an ethnicity question. Collectively, 3.4% of these participants (*n* = 373) identified as Aboriginal and Torres Strait Islander people, however across the latter two question formats (i.e., excluding the sole study of *n* = 336 participants who were all Aboriginal and Torres Strait Islander people), less than 1% of participants (0.6%, approximate *n* = 62) identified as Aboriginal or Torres Strait Islander. Of the five studies (*n* = 6,843 participants) that provided data on ethnicity, the majority of participants identified solely as “Caucasian” (*n* = 6,615; 96.7%).

Seven studies (*n* = 7,679 participants) provided data on relationship status. Of these, 68.2% (*n* = 5,240) of participants were partnered (married/living with a partner/de‐facto), 11.3% (*n* = 865) were widowed, 10.3% (*n* = 794) were divorced or separated, 7.9% (*n* = 609) were never married/single, and 2.2% (*n* = 171) were classified as other/unsure.

Participant postcode or pre‐specified rurality classification data were provided by five studies (*n* = 15,604 participants). Based on Modified Monash Model (MMM) classifications, most participants (44.8%) were from a regional center (MMM = 2, *n* = 6,994) or major city (MMM = 1; 24.2%, *n* = 3,780), whilst 17.9% were from small rural towns (MMM = 5, *n* = 2,797). Only 2.7% of participants were from remote or very remote locations (MMM 6 or 7, *n* = 426).

Six studies provided data on either first language (*n* = 4, 1,925 participants) and/or preferred/primary language (*n* = 4, 1,718 participants). The majority of participants reported English as their first language (96.4%, *n* = 1,855), and similarly, most reported English as their primary or preferred language (95.9%, *n* = 1,648).

Eight studies provided data on at least one indicator of socio‐economic status (*n* = 17,390 participants). Of these, six studies provided data on main occupation, four studies provided data on living arrangements, one study provided data on IRSAD decile, and two studies provided data on income or income‐related information (e.g., “How well do you manage with the income you have available?”). Due to the heterogeneity in variable format and type, they were not able to be harmonized for summary across studies.

No studies provided data on participant nationality or research team characteristics and recruitment approaches. We note that one study did provide data on sexual preference (options: “males,” “females,” “both,” “not interested in sex,” “prefer not to answer”), but were not analyzed as “sexual orientation” to avoid making assumptions based on participant's reported sex and/or gender identity.

### 3.4 Vision workshop

#### 3.4.1 Reflection on current practices

**FIGURE 2:**
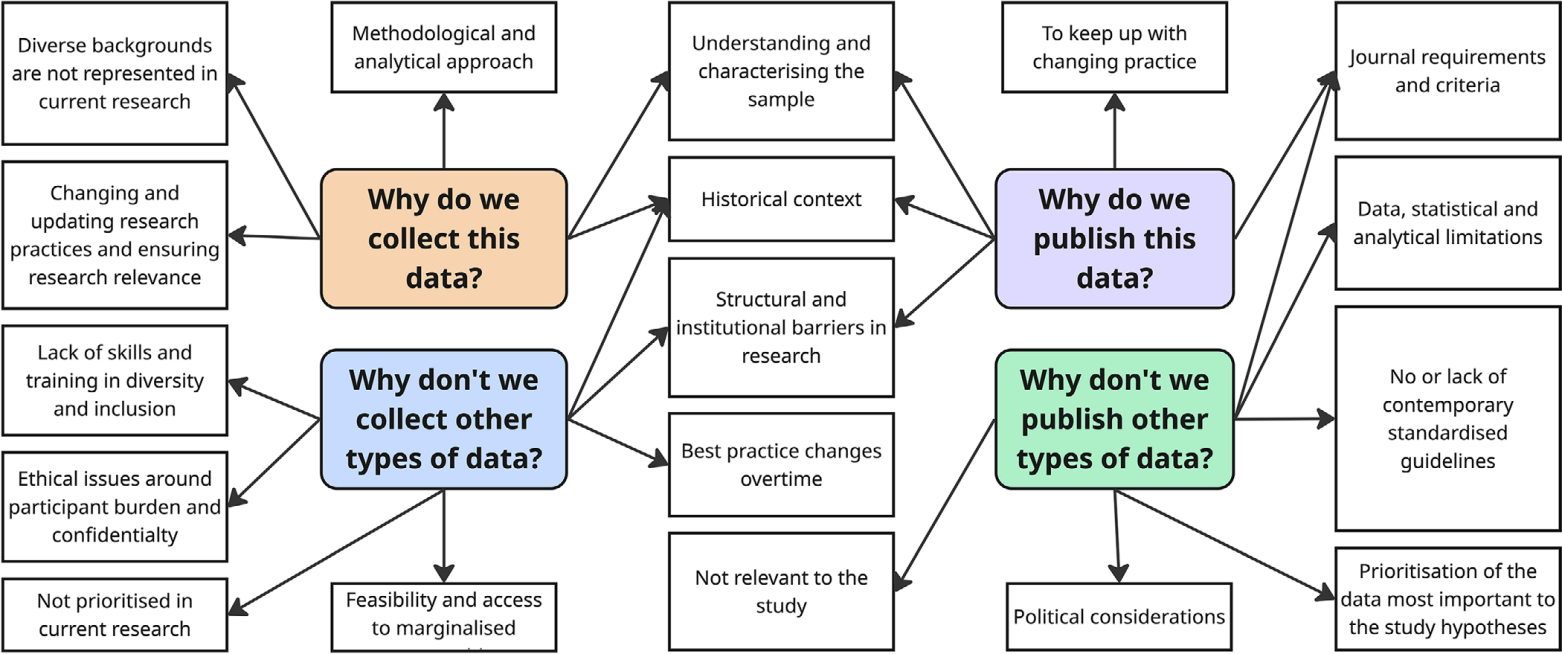
Main themes arising from each of the four questions (in coloured text boxes) asked during workshop Task 1. Themes identified through thematic analysis are presented in white boxes.

Eighteen themes were identified from responses in Task 1 (Figure 2). For Question 1, “Why do we collect this demographic data?”, key themes were: historical context (e.g., “it is the data which has always been collected”); necessary to methodological analyses/approaches; to understand and characterize the sample; due to changing research practices to ensure research relevance; and because diverse backgrounds are not represented. For Question 2, “Why do we publish this data?”, the main themes included were: to characterize the sample and contextualize factors; due to journal requirements; structural and institutional barriers in research; historical context; and changing practice. Key themes for Questions 3 and 4, “Why don't we collect/publish other types of data?”, included: lack of training around diversity and inclusion, feasibility of access to (and working with) marginalized communities, lack of standardized reporting frameworks, and data or statistical limitations.

#### 3.4.2 Consequences of current practices and changes to practice

**FIGURE 3:**
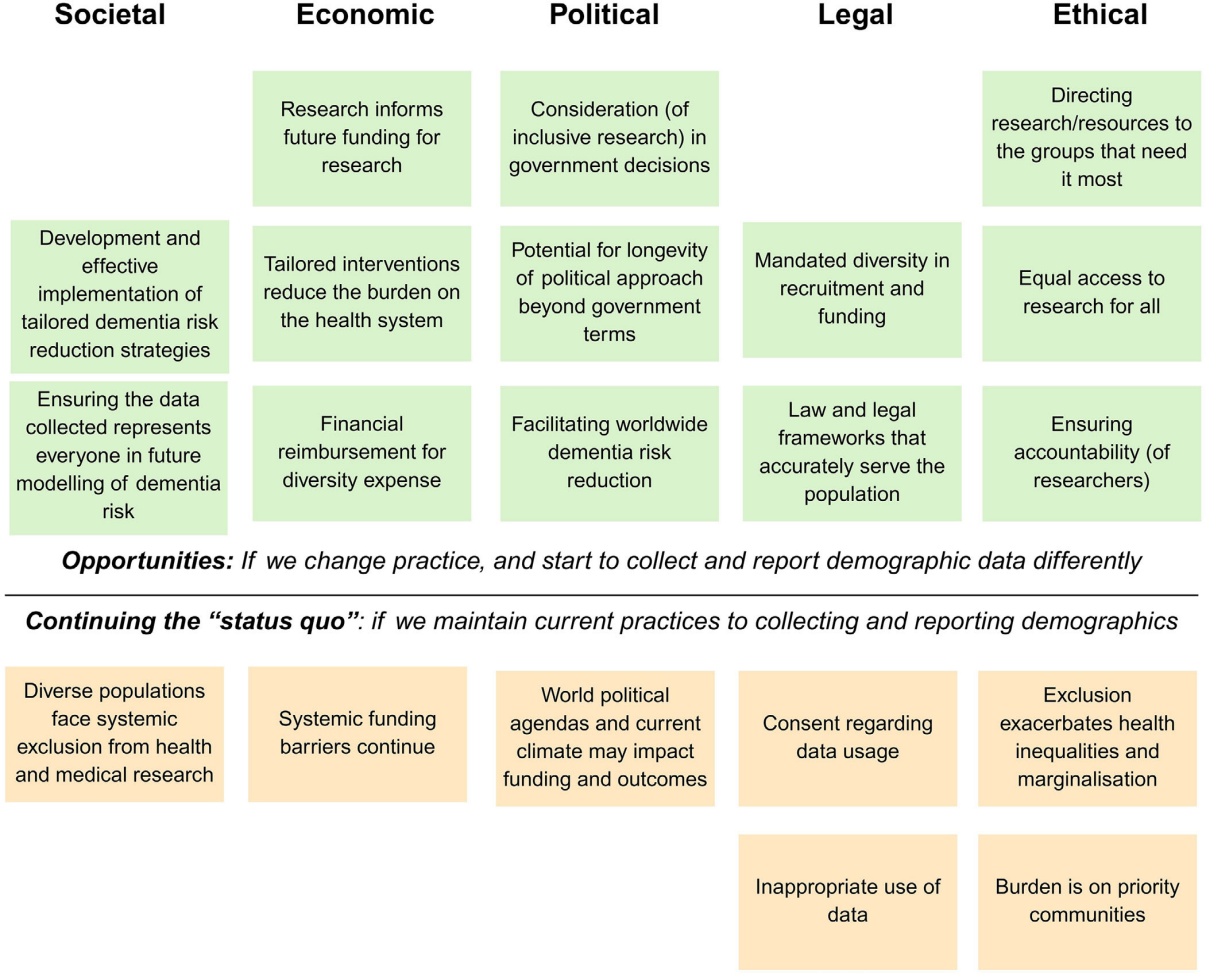
Outcomes of Tasks 2 and 3 in the Vision workshop. The consequences of continuing to collect and publish demographic data as is currently done (i.e., “continuing the status quo”) are indicated in yellow text boxes. Opportunities that may arise from improving demographic data collection and reporting practices are indicated in green text boxes. Consequences are grouped into societal, economic, political, legal, and ethical perspectives (top headers) in line with workshop prompts.

In Tasks 2 and 3, participants were asked to consider why their responses (i.e., the reasons for collecting and publishing demographic data, or not) matter from a social, economic, political, legal, and ethical perspective. Then, they were asked what the future consequences might be if we maintain the status quo, or conversely, make substantial changes to practice (Figure 3). Potential barriers to change were identified, such as system funding barriers negatively impacting the ability to conduct research in collaboration with priority populations. The potential negative ramifications of collecting more demographic data also included the potential for inappropriate data usage. Key identified opportunities included the ability to more effectively model dementia risk in a way that is tailored to the individual, and to ensure equal access to the benefits of research for all. From an ethical perspective, it was noted that being transparent in the collection and publication of demographic data encourages accountability to ensure researchers have made intentional efforts to include priority populations.

#### 3.4.3 Recommendations generated by researchers

Thirty‐nine recommendation statements developed from the final workshop task were reviewed by workshop attendees in the follow up survey. A total of 25 participants (of the people that were initially invited to the vision workshop) responded to the survey. Two recommendations received more than two “veto” votes and were excluded (one each from the government and institution sections). Supplementary File 9 contains a full list of recommendations across government, institution and researcher levels, and associated statistics (rank, number of whole‐hearted endorsements, and agreement between experts). The recommendations are firstly ordered by rank and then by number of whole‐hearted endorsements, therefore recommendations nearer to the top can be interpreted as higher priority for immediate action, with priority declining thereafter.

At the government level, recommendations for systemic change (Table 1) included supporting greater representation of underrepresented communities in research (and as the focus of research), on health advisory boards, as collaborators in research development, and across the field by encouraging participation and retention of researchers from these communities (Recommendations 1, 2, 5, 6, and 8). Appropriate funding to strengthen connections with and conduct research in diverse communities was encouraged (Recommendations 4 and 7). Further government‐level recommendations focused on developing policies and providing centralized support for the collection, sharing and reporting of detailed demographic data (Recommendations 3, 9, 10, and 13). The most strongly endorsed recommendation receiving the highest percentage of whole‐hearted endorsement (72% of researchers surveyed) was Recommendation 2: *policies related to dementia prevention and risk reduction must be developed through engagement with the target population and must live beyond the political term*.

**TABLE 1:** Recommendations for government‐level change to improve recruitment and reporting practices in dementia prevention research.

Rank	Recommendations/priorities
1	Ensure there is a focus on diversity and social determinants of health in medical and scientific research
2	Policies related to dementia prevention and risk reduction must be developed through engagement with the target population and must live beyond the political term
3	Have a minimum list of demographics which should be collected in government‐funded dementia risk reduction research
4	Funding bodies should develop specific grant rounds focused on diversity in dementia research and follow up on sample representativeness, specifically focusing on population‐representative cohort studies and taking into account factors such as the track record of diversity in the previous samples of the Chief Investigator
5	Focus on systemic change to allow individuals from under‐represented communities to engage in research: teaching the importance of diversity in schools, providing specific opportunities for diverse groups to engage in research (including access to university)
6	Have a greater representation of diverse populations on health advisory boards
7	Dedicate funding to building relationships with diverse communities, both prior to the start of the research and during the project development phase
8	Eliminate barriers that diverse groups face when volunteering for research
9	Support data linkage and communication between governments, research institutions, and researchers—make this process easier practically and financially
10	Have more centralized support for researchers to develop diversity measures into their research
11	Develop policies which protect data collection to facilitate collection of rich demographic data
11	Identify which groups are missing from census data and national‐level data sets
12	Develop policies in conjunction with researchers through systematic reviews and meta‐analyses

*Note*: Recommendations were generated by Australian dementia researchers and ranked using a multiple criteria decision analysis (ordered by highest rank and then by number of whole‐hearted endorsements for recommendations with equal rank).

At the institutional level (e.g., universities, societies, or industry‐based research centers; Table 2), recommendations focused on increasing support for diverse communities through diversity awareness training for researchers, development of committees to assist with conducting research on and with priority populations, and researcher collaborations (Recommendations 2, 3, 4, and 6). In addition, there was a focus on the procedural side of research, including urging ethics committees to focus on and monitor participant diversity, increasing the ease of data sharing, and encouraging open access frameworks (Recommendations 8, 9, and 13). Recommendations also included the development of institution‐specific guidelines for recruitment of participants and reporting of demographic data (Recommendations 1 and 5). Funding constraints were highlighted, with recommendations to ensure sufficient funding for sub‐group analyses and to extend studies with additional follow‐ups (Recommendations 12 and 7). We note that, due to the broad definition of “institution” used here, these recommendations cut across a diversity of institutions and may require addressing at the broader research ecosystem level. The recommendation which received the highest percentage of whole‐hearted endorsement (60%) was Recommendation 1: *Institutions and journals should develop reporting guidelines for diversity in research, making it the gold standard to collect a broad range of demographic data, and developing standardized terms for improved harmonization of data (both within Australia and internationally)*.

**TABLE 2:** Recommendations for institution‐level change to improve recruitment and reporting practices in dementia prevention research.

Rank	Recommendations/priorities
1	Institutions and journals should develop reporting guidelines for diversity in research, making it the gold standard to collect a broad range of demographic data, and developing standardized terms for improved harmonization of data (both within Australia and internationally)
2	Improve diversity awareness and training for researchers, including asking Chief Investigators to complete implicit bias testing, and having this training implemented much earlier in their career (i.e., in undergraduate years)
3	Increase support to encourage retention of researchers from diverse communities
4	Institutions should form better recruitment policies and procedures, including reviewing these policies to identify barriers to diverse recruitment
5	Develop committees and teams dedicated to diversity, where representatives are appropriately re‐imbursed for their time and expertise. This committee could assist with employing researchers with lived experience to collect data
6	Facilitate opportunities for collaboration with communities and between research from a diverse range of backgrounds
7	Continue to fund studies beyond their original terms as a mechanism to continue legacy cohort studies
8	Ethics applications should have a bigger focus on diversity as an ethical issue. Increase check‐ins along the way and be more flexible around different consent procedures for inclusion of underrepresented populations
9	Increase the ease of data‐sharing agreements between institutions
10	Emphasise the holistic, life‐course conceptualization of dementia, including a greater integration on social models of health
11	Make efforts to understand different procedures and knowledge which look beyond 'Western' paradigms
12	Ensure there is sufficient funding for meaningful investigation into sub‐group analyses
13	Journals should increase support for opinion pieces and commentaries encouraging discourse, and for studies which highlight systemic issues to the government
14	Encourage open access data frameworks/policies from the beginning

*Note*: Recommendations were generated by Australian dementia researchers and ranked using a multiple criteria decision analysis (ordered by highest rank and then by number of whole‐hearted endorsements for recommendations with equal rank).

Recommendations for individual researchers (Table 3) included reflecting on the way personal biases and behavior can influence one's own research, as well as influence the standards set in the community, and being proactive to change these patterns (Recommendations 2, 8, and 10). Intentionality in the allocation of resources, selection of assessment tools and methods of recruitment was urged (Recommendation 5, 6, and 7). Additionally, it was recommended that researchers make efforts to develop connections with the communities they are researching and to collaborate with researchers who have expertise relevant to the priority population (Recommendations 1 and 3). The recommendation which received the highest percentage of whole‐hearted endorsement (64%) was Recommendation 1: *Make efforts to develop connections with the communities you are researching*.

**TABLE 3:** Recommendations for individual researcher‐level change to improve recruitment and reporting practices in dementia prevention research.

Rank	Recommendations/priorities
1	Make efforts to develop connections with the communities you are researching
2	Assess and address personal and positional biases in your knowledge and experience. Engage in the necessary trainings to address these
3	Collaborate with researchers who have expertise in researching diverse populations, those with lived experience and researchers beyond the “dementia bubble”. Increase co‐design practices, build in diversity from the beginning of the project and not as an afterthought
4	Budget and resource projects properly to facilitate inclusion, including paying researchers for their time and expertise
5	Be transparent in communicating the diversity of your sample
6	Carefully consider the tools being used to assess your sample. Choose tools which are inclusive
7	Be intentional about recruitment methodologies
8	Keep up with and help to pioneer the gold standard of demographic data collecting and reporting
9	Support/mentor diverse students
10	Work to support a change in mindset for diversity to be at the forefront of all research
11	Use the data you already have to investigate samples and empirically demonstrate the power of representative samples, including collaboration on cohort studies

*Note*: Recommendations were generated by Australian dementia researchers and ranked using a multiple criteria decision analysis (ordered by highest rank and then by number of whole‐hearted endorsements for recommendations with equal rank).

## 4 DISCUSSION

This study aimed to evaluate the diversity and inclusivity of Australian dementia prevention research. To achieve this, the demographic characteristics of participants and reporting practices of such characteristics in Australian longitudinal cohort and intervention studies were evaluated in a two‐stage synthesis, using both published cohort‐level data, and as a supplementary analysis, de‐identified participant‐level data submitted by primary data custodians. Secondly, we aimed to generate recommendations to inform recruitment and improve the representativeness of future dementia prevention research by presenting the findings from Stage 1 to Australian dementia prevention researchers through an interactive workshop.

### 4.1 Trends in data collection and reporting practices

Our findings highlight several patterns in reporting practices in Australian dementia prevention research. Among the studies identified in the systematic literature search, age, sex or gender, education level, and geographical location of participants were the most commonly reported demographic characteristics, whereas variables related to cultural and linguistic diversity and sexual orientation of participants were the least reported. Although many variables were reported using consistent (or reasonably similar) approaches, some were difficult to synthesize across studies (e.g., educational attainment/highest qualification, socio‐economic status) due to differences in measurement, data collection and classification. No studies reported both the sex and gender of their participants; however, all studies reported at least one of the two variables. Further, the discrepancy in language used to refer to sex and gender was a barrier in the synthesis and comparison of these variables.

### 4.2 Representation and generalizability of Australian dementia prevention research

Several of the demographic characteristics captured in this review do not align with trends in the general Australian population. Among the published studies which provided years of education data (mean age ∼68 years), 75% of participants had completed more than 12 years of education. In contrast, the Australian Institute of Health and Welfare estimates approximately 31% of Australians aged >65 years reported completing a certificate or diploma‐level qualification or higher (including tertiary education) in 2016.^64^ Among the submitted participant‐level data (from studies that did not recruit solely Aboriginal and Torres Strait Islander participants), less than 1% of participants identified as Aboriginal and Torres Strait Islander peoples, whilst it is estimated that 3.8% of the total Australian population are Aboriginal and Torres Strait Islander peoples.^65^ Over 90% of participants in this review reported English as their primary or preferred language, compared to approximately 77% of the Australian population (who indicated only speaking English at home).^66^ Finally, only 0.03% of participants (*n* = 9) included in this review identified their gender as “other” (i.e., not man or woman), with no further clarification of their specific gender identity, and no data available on sexual orientation. In contrast, an estimated 910,600 Australians aged >16 years identify as LGBTI+, with approximately one in five of these Australians identifying as transgender and/or gender diverse. Approximately 8% of LGBTI+ Australians are aged over 65 years (i.e., ∼72,000 people^67^). This under‐representation may reflect that these data were not collected (i.e., the response options for gender were “man” or “woman” only in some studies). However, it cannot be ruled out that Australian dementia prevention research is not adequately sampling participants from these groups.

Taken together, our findings suggest that participants being recruited into Australian dementia prevention cohort and intervention studies are more educated and less diverse in terms of culture, language, ethnicity and gender than the general Australian population. We acknowledge that some of these comparisons are based on the total Australian population (rather than those aged >65 years), and that comparison to demographic characteristics obtained through large population‐based surveys (i.e., census data), particularly characteristics relating to cultural and linguistic diversity, may be limited by the potential biases in question formats and data collection materials used in these surveys.^68^ However, this also indicates potential broader issues with respect to restricted data collection in surveys which characterize the Australian population. Additionally, it is important to note that some studies captured in this review were initiated over two decades ago (*n =* 5 published studies, 12,762 participants), thus the demographic characteristics of their sample likely do not align with the modern Australian population.

The under‐representation of minoritized groups is common across dementia research both globally and within Australian settings,^20^ and a key contributor to the limited representation of these communities in dementia research is the approach taken to recruit participants.^21^, ^22^, ^69^ In the current study, recommendations generated during the vision workshop across all three levels (government, institution, individual) reflected concerns surrounding the lack of inclusion and representation of Aboriginal and Torres Strait Islander peoples and CALD communities in particular. For example, researchers highlighted the importance of making efforts and providing opportunities for engaging and building connections with the communities involved in the research (government—Recommendation 7; institutions—Recommendation 6; individual researchers—Recommendations 1 and 3). Making an intentional effort to ensure inclusivity of underrepresented communities includes carefully considering the tools being used for data collection (individual researchers—Recommendation 6) and assessing personal biases (of the researcher) and engaging in necessary training to address these (individual researchers—Recommendation 2). These recommendations echo previous work among CALD communities, highlighting that key influencing factors of trial participation include using inclusive recruitment strategies (including partnering with communities and community leaders or representatives), translating trial materials into languages other than English (and adapting content to other cultures), and promoting retention via building rapport with clear and culturally relevant explanations of research processes.^70^, ^71^

To facilitate evidence‐based practice and ensure policy guidelines are based on representative data, the Australian Government implemented the CALD Dementia Research Action Plan in 2019^72^ and recently released their National Dementia Action Plan for 2024‐2034.^73^ Within this 10‐year plan, the inclusion of Aboriginal and Torres Strait Islander peoples and CALD communities at all stages of publicly funded research projects was urged.^73^ It was also recommended that evidence‐based risk reduction interventions, targeted strategies, and health messages for people from diverse communities and those at higher risk of developing dementia must be developed in partnership with these communities.^73^ Indeed, co‐designing or co‐producing research with priority populations is considered best‐practice to overcome disparities and inequities,^22^, ^74^, ^75^ Whilst there are frameworks and necessary actions occurring at the government level, including ongoing national funding schemes to address dementia prevention and health inequalities (including those searched in Phase 1), the current work highlights recommendations for actions and areas for improvement that can continue to be addressed at the institutional and individual level.

### 4.3 Strengths and limitations

The current study used a comprehensive multi‐stage mixed‐methods design which allowed thorough exploration of the demographic characteristics of participants involved in Australian dementia prevention research. Efforts were made to locate the cohort profiles of the identified studies to ensure the most accurate representation of each cohort or intervention study was provided. Recognizing that many studies collect more data than they publish, we also reached out to primary authors and data custodians for access to all de‐identified demographic variables to include in a supplementary analysis. Additionally, we used a participatory co‐design method during vision workshops and collected feedback and recommendations from Australian dementia prevention researchers using participant‐led documentation (rather than academic scribing) to reduce bias. Themes derived by the core research team were communicated back to all contributors to confirm agreement with recommendations generated (or otherwise).

Nine cohort studies and five intervention studies were able to share de‐identified participant‐level demographic data with the research team for synthesis, representing 60% of identified cohort studies but only 38% of RCTs. This disparity may suggest that cohort studies are currently better positioned to support collaborative data sharing, highlighting both the strength of the current study and potential areas for growth in intervention data practices. Despite willingness for data sharing from many data custodians, the time, ethical and legal complexity of establishing data sharing agreements posed a major barrier. Although all data sharing agreements were initiated between July 2024 and December 2024, some took more than 12 months to fully execute by all parties. Such delays hinder progress by limiting collaboration across institutions and preventing the efficient analysis of existing data for new research questions. The difficulty of data sharing was also raised by researchers in the vision workshop, reflected in Recommendation 9 for institutions (increase the ease of data‐sharing agreements between institutions). As a result, the participant‐level data presented here represent only a small snapshot of broader data available across Australian dementia prevention research.

The published cohort‐level datasets included in this synthesis varied widely in year of study initiation, with some studies initiated in the early 1990s. Studies were not analyzed separately by year of study initiation as several of these earlier datasets are still being used to generate evidence on dementia prevention currently. However, it is important to acknowledge that, although the demographic profiles of these earlier studies may not align with the modern Australian population, they may have been representative at the time of initiation. Thus, our comparisons between dementia prevention research samples and the general Australian population should be interpreted with this in mind.

We synthesized the frequency and distribution of demographic characteristics separately in the participant‐level data synthesis to reduce the potential for re‐identifiability of participants involved (particularly those from minoritized groups). However, it must be acknowledged that demographic characteristics do not exist in silos, and the intersectionality of such characteristics can greatly exacerbate individuals’ susceptibility to modifiable dementia risk factors, and opportunities to reduce their dementia risk. Assessing the representation of groups who exist at the intersection of multiple demographic subgroups (e.g., older, gender diverse and CALD individuals) is an important future direction for this field.

Importantly, whilst recommendations for future work were generated by Australian dementia prevention researchers with diverse expertise and training backgrounds, data were not gathered about the demographic characteristics (and diversity) of researchers involved in the workshop. Thus, the possibility that researchers’ personal biases and viewpoints may not reflect those of all researchers at the center of this work must be acknowledged.

### 4.4 Future directions

To advance the representation of minoritized communities in dementia prevention research, several recommendations were identified at government, institutional, and individual researcher levels. At the government level, policies related to dementia prevention and risk reduction must be developed in partnership with the target population and must live beyond the political term. At the institutional level, clear and dynamic guidelines for the reporting and sharing of demographic data (e.g., gender, sex, and sexual orientation terminology) should be established to ensure consistency and transparency across studies. These guidelines should be developed in collaboration with non‐academic end‐users and advisory groups which represent minoritized groups through further co‐design, extending beyond the voice and perspectives of research academics alone. Importantly, such future guidelines must be considerate of geographical and cultural contexts which may impact the format and type of demographic data that can be safely collected and shared. Another area of focus at the institutional level should be the development of streamlined processes for data sharing agreements to reduce the delay in accessing and analyzing already collected data. At the individual researcher level, training, support, and research funding are needed to enable meaningful engagement with underserved communities, including navigating the ethical, legal, and practical processes for collecting sensitive and potential re‐identifiable demographic information. Together, these measures will help ensure future dementia prevention research and the guidelines and policies which are generated are representative of the diverse Australian population and are inclusive, representative, and capable of addressing diverse population needs.

### 4.5 Conclusion

The findings of this mixed‐methods review suggest that participants being recruited into Australian dementia prevention cohort and intervention studies do not accurately reflect the cultural, ethnic, linguistic and gender diversity of the contemporary Australian population. Dementia prevention research that accurately reflects the Australian population is imperative to identify risk factors which disproportionately affect priority populations, and to facilitate the development of interventions which are targeted, effective, and culturally and contextually appropriate. Increasing representation in research may also help to identify priority areas in current systems (e.g., health care) that need to be addressed to more effectively reduce dementia risk in the community. Together, these efforts are crucial to reducing the disproportionate impact of dementia on minoritized communities in Australia. While the new National Dementia Action Plan^73^ directly references diverse populations, the present research identifies opportunities at all levels (government, institution and individual researcher) to ensure Australian dementia prevention research truly meets the needs of the increasingly diverse (and dynamic) Australian population. The recommendations presented in this paper provide achievable and actionable changes which can be made both by individual researchers and broader institution or government‐level policymakers to facilitate inclusive and representative dementia prevention research in Australia.

## CONFLICT OF INTEREST STATEMENT

The authors declare no conflicts of interest relevant to this article. A.B.F. acts as the Director of Neuropsychology for NewDays.ai; however, this work is unrelated to the topic of the current manuscript. Author disclosures are available in the Supporting Information.

## Supporting information




Supporting Information

Supporting Information

Supporting Information

Supporting Information

Supporting Information

Supporting Information

Supporting Information

Supporting Information

Supporting Information

Supporting Information
