# Supplementary material for: Diversity and inclusivity in Australian dementia prevention research: A mixed methods review
Source: Alzheimers Dement (N Y). 2026 Jul 18;12(3):e70296. doi: 10.1002/trc2.70296 (PMC13380669; doi:10.1002/trc2.70296)
Supplement: Supplementary file 2 — Supporting Information [file TRC2-12-e70296-s004.docx]

## **Supplementary File 2**

## Search strategy

## **Search 1: completed on 31.10.2023**

**Medline (Ovid)**

| **Search number** | **Search terms** | **Search results** |
| --- | --- | --- |
| 1 | dementia.mp. or Dementia, Vascular/ or exp Dementia/ or Frontotemporal Dementia/ or Dementia, Multi-Infarct/ | 264,542 |
| 2 | Cognitive Dysfunction/ or Cognition Disorders/ or Alzheimer Disease/ | 205,931 |
| 3 | 1 or 2 | 332,276 |
| 4 | risk reduction.mp. or Risk Reduction Behavior/ | 36,286 |
| 5 | prevention.mp. | 1,939,679 |
| 6 | 4 or 5 | 1,955,727 |
| 7 | exp Australia/ | 171,920 |
| 8 | clinical study/ or clinical trial/ or clinical trial protocol/ or observational study/ or comparative study/ or evaluation study/ or meta-analysis/ or "systematic review"/ or validation study/ | 3,054,746 |
| 9 | exp cohort studies/ or controlled before-after studies/ or cross-sectional studies/ or interrupted time series analysis/ | 2,922,887 |
| 10 | 8 or 9 | 5,325,245 |
| 11 | 3 and 6 and 7 and 10 | 101 |

**EMBASE (Ovid)**

| **Search number** | **Search terms** | **Search results** |
| --- | --- | --- |
| 1 | semantic dementia/ or mixed dementia/ or dementia assessment/ or exp dementia/ or clinical dementia rating scale/ or dementia.mp. or Cornell Scale for Depression in Dementia/ or Mattis Dementia Rating Scale/ or frontal variant frontotemporal dementia/ or Pick presenile dementia/ or Blessed dementia scale/ or senile dementia/ or "mixed depression and dementia"/ or multiinfarct dementia/ or frontotemporal dementia/ or presenile dementia/ or HIV associated dementia/ | 491,374 |
| 2 | cognitive defect/ or mild cognitive impairment | 248,064 |
| 3 | 1 or 2 | 648,966 |
| 4 | risk reduction/ | 131,892 |
| 5 | prevention/ | 325,279 |
| 6 | 4 or 5 | 455,418 |
| 7 | exp Australia/ | 206,935 |
| 8 | exp clinical protocol/ or exp clinical trial protocol/ | 122,603 |
| 9 | meta analysis/ | 294,228 |
| 10 | “systematic review”/ | 435,921 |
| 11 | exp follow up/ or exp major clinical study/ or exp cohort analysis/ | 6,756,226 |
| 12 | exp cross-sectional study/ | 587,918 |
| 13 | 8 or 9 or 10 or 11 or 12 | 7,601,914 |
| 14 | 3 and 6 and 7 and 13 | 57 |

**PsycINFO (Ovid)**

| Search number | Search terms | Search results |
| --- | --- | --- |
| 1 | Dementia with Lewy Bodies/ or Senile Dementia/ or exp Dementia/ or Semantic Dementia/ or dementia.mp. or Presenile Dementia/ or Vascular Dementia/ | 118,003 |
| 2 | exp Cognitive Impairment/ | 44,818 |
| 3 | 1 or 2 | 144,632 |
| 4 | Prevention/ or exp Risk Assessment/ or exp At Risk Populations/ or exp Risk Factors/ | 185,645 |
| 5 | australia.mp. [mp=title, abstract, heading word, table of contents, key concepts, original title, tests & measures, mesh word] | 47,931 |
| 6 | exp Clinical Trials/ or exp Intervention/ | 154, 688 |
| 7 | protocol.mp. [mp=title, abstract, heading word, table of contents, key concepts, original title, tests & measures, mesh word] | 41,682 |
| 8 | cohort studies.mp. | 31,359 |
| 9 | cross-sectional studies.mp. | 71,648 |
| 10 | 6 or 7 or 8 or 9 | 289,120 |
| 11 | 3 and 4 and 5 and 10 | 33 |

**CINAHL (EBSCO)**

| Search number | Search terms | Search results |
| --- | --- | --- |
| 1 | "Dementia/SS/RF/PF/PC/PA/MO/FG/EP/ET/DI/CL") OR "dementia" OR (MH "Frontotemporal Dementia") OR (MH "Dementia, Vascular") OR (MH "Delirium, Dementia, Amnestic, Cognitive Disorders") OR (MH "Dementia, Multi-Infarct") OR (MH "Lewy Body Disease") OR (MH "Dementia, Presenile") OR (MH "Dementia, Senile") OR (MH "Dementia Patients") | 78,607 |
| 2 | (MH "Mild Cognitive Impairment") OR (MH "Cognition Disorders") OR "cognitive impairment or cognitive dysfunction or cognitively impaired" OR (MH "Memory Impairment (Saba CCC)") OR (MH "Impaired Memory (NANDA)") | 35,975 |
| 3 | S1 OR S2 | 106,202 |
| 4 | Prevention | 777,523 |
| 5 | Risk reduction | 10,194 |
| 6 | S4 OR S5 | 782,112 |
| 7 | (MH "Australia+") OR "Australia" OR (MH "Western Australia") OR (MH "South Australia") OR (MH "Victoria") OR (MH "Tasmania") OR (MH "Queensland") OR (MH "Northern Territory") OR (MH "New South Wales") | 146,495 |
| 8 | Protocol | 103,818 |
| 9 | (MH "Prospective Studies") OR "cohort profile" | 528,576 |
| 10 | (MH "Cross Sectional Studies") OR "cross sectional study" OR (MH "Controlled Before-After Studies") | 272,983 |
| 11 | S8 OR S9 OR S10 | 859,720 |
| 12 | S3 AND S6 AND S7 AND S11 | 71 |

# **Clinical Trials Registry**

## **Search on 13.11.2023**

**ANZCTR**

| Registry | ANZCTR |
| --- | --- |
| Description of intervention(s) / exposure | N/A |
| Intervention code: OR | - Early detection/Screening - Prevention - Lifestyle - Behaviour - Other interventions |
| Study type | N/A |
| Allocation to intervention | N/A |
| Recruitment status | Completed |
| Health condition(s) or problem(s) studied | Dementia |
| Condition category | N/A |
| Gender | N/A |
| Age group | N/A |
| Ethics application status | N/A |
| Healthy volunteers | N/A |
| Registration date | N/A |
| Trial start date | N/A |
| Countries of recruitment | Australia |
| Location within Australia | N/A |
| Primary sponsor type | N/A |
| Funding source type | N/A |
| Phase | N/A |
| Search results: 46 | |

Full list of articles downloaded, only added to literature excel spreadsheet following screening

**clinicaltrials.gov**

| Condition/disease | Dementia OR Alzheimer's Disease OR Cognitive Impairment |
| --- | --- |
| Other terms | Prevention OR Risk Reduction OR Protocol |
| Intervention/treatment | N/A |
| Location | Australia |
| Study Status | Looking for participants (Recruiting)  No longer looking for participants (Active, not recruiting and completed) |
| Sex | All |
| Age | All |
| Study phase | N/A |
| Study type | N/A |
| Study results | N/A |
| Study documents | N/A |
| Funder type | N/A |
| Date range | N/A |
| More ways to search | N/A |
| Search results: 23 | |

**CENTRAL (through the Cochrane library)**

| Search number | Search terms | Search results |
| --- | --- | --- |
| 1 | "Dementia" OR "Alzheimer's Disease" OR "Cognitive Impairment" (Title Abstract Keyword) | 31161 |
| 2 | "Prevention" OR "Risk reduction" (All Text) | 230280 |
| 3 | “Australia” (All Text) | 44370 |
| 4 | "Protocol" OR "Cohort studies" OR "Clinical Trial" (All Text) | 588716 |
| 5 | #1 AND #2 AND #3 AND #4 | 145 |

## **Search 2: completed on 11.02.2025**

**Search period: 31.10.2023 - 11.02.2025**

**Medline (Ovid)**

| **Search number** | **Search terms** | **Search results** |
| --- | --- | --- |
| 1 | dementia.mp. or Dementia, Vascular/ or exp Dementia/ or Frontotemporal Dementia/ or Dementia, Multi-Infarct/ | 287318 |
| 2 | Cognitive Dysfunction/ or Cognition Disorders/ or Alzheimer Disease/ | 222715 |
| 3 | 1 or 2 | 359567 |
| 4 | risk reduction.mp. or Risk Reduction Behavior/ | 38600 |
| 5 | prevention.mp. | 2049301 |
| 6 | 4 or 5 | 2066632 |
| 7 | exp Australia/ | 181143 |
| 8 | clinical study/ or clinical trial/ or clinical trial protocol/ or observational study/ or comparative study/ or evaluation study/ or meta-analysis/ or "systematic review"/ or validation study/ | 3160245 |
| 9 | exp cohort studies/ or controlled before-after studies/ or cross-sectional studies/ or interrupted time series analysis/ | 3143127 |
| 10 | 8 or 9 | 5618640 |
| 11 | 3 and 6 and 7 and 10 | 116 |
| 12 | limit 11 to yr="2023 -Current" | 22 |
| 13 | from 12 keep 1-13 | 13 |

**EMBASE (Ovid)**

| **Search number** | **Search terms** | **Search results** |
| --- | --- | --- |
| 1 | semantic dementia/ or mixed dementia/ or dementia assessment/ or exp dementia/ or clinical dementia rating scale/ or dementia.mp. or Cornell Scale for Depression in Dementia/ or Mattis Dementia Rating Scale/ or frontal variant frontotemporal dementia/ or Pick presenile dementia/ or Blessed dementia scale/ or senile dementia/ or "mixed depression and dementia"/ or multiinfarct dementia/ or frontotemporal dementia/ or presenile dementia/ or HIV associated dementia/ | 531587 |
| 2 | cognitive defect/ or mild cognitive impairment | 280109 |
| 3 | 1 or 2 | 707821 |
| 4 | risk reduction/ | 138899 |
| 5 | prevention/ | 350531 |
| 6 | 4 or 5 | 487442 |
| 7 | exp Australia/ | 217511 |
| 8 | exp clinical protocol/ or exp clinical trial protocol/ | 130069 |
| 9 | meta analysis/ | 342253 |
| 10 | “systematic review”/ | 509735 |
| 11 | exp follow up/ or exp major clinical study/ or exp cohort analysis/ | 7388359 |
| 12 | exp cross-sectional study/ | 689902 |
| 13 | 8 or 9 or 10 or 11 or 12 | 8347252 |
| 14 | 3 and 6 and 7 and 13 | 67 |
| 15 | limit 14 to yr="2023 -Current" | 16 |
| 16 | from 15 keep 2-7, 11, 14, 16 | 9 |

**PsycINFO (Ovid)**

| Search number | Search terms | Search results |
| --- | --- | --- |
| 1 | Dementia with Lewy Bodies/ or Senile Dementia/ or exp Dementia/ or Semantic Dementia/ or dementia.mp. or Presenile Dementia/ or Vascular Dementia/ | 126812 |
| 2 | exp Cognitive Impairment/ | 48395 |
| 3 | 1 or 2 | 155510 |
| 4 | Prevention/ or exp Risk Assessment/ or exp At Risk Populations/ or exp Risk Factors/ | 198514 |
| 5 | australia.mp. [mp=title, abstract, heading word, table of contents, key concepts, original title, tests & measures, mesh word] | 51121 |
| 6 | exp Clinical Trials/ or exp Intervention/ | 165671 |
| 7 | protocol.mp. [mp=title, abstract, heading word, table of contents, key concepts, original title, tests & measures, mesh word] | 46214 |
| 8 | cohort studies.mp. | 32485 |
| 9 | cross-sectional studies.mp. | 74688 |
| 10 | 6 or 7 or 8 or 9 | 308083 |
| 11 | 3 and 4 and 5 and 10 | 35 |
| 12 | limit 11 to yr="2023 -Current" | 1 |

**CINAHL (EBSCO)**

| Search number | Search terms | Search results |
| --- | --- | --- |
| 1 | "Dementia/SS/RF/PF/PC/PA/MO/FG/EP/ET/DI/CL") OR "dementia" OR (MH "Frontotemporal Dementia") OR (MH "Dementia, Vascular") OR (MH "Delirium, Dementia, Amnestic, Cognitive Disorders") OR (MH "Dementia, Multi-Infarct") OR (MH "Lewy Body Disease") OR (MH "Dementia, Presenile") OR (MH "Dementia, Senile") OR (MH "Dementia Patients") | 82109 |
| 2 | (MH "Mild Cognitive Impairment") OR (MH "Cognition Disorders") OR "cognitive impairment or cognitive dysfunction or cognitively impaired" OR (MH "Memory Impairment (Saba CCC)") OR (MH "Impaired Memory (NANDA)") | 38310 |
| 3 | S1 OR S2 | 111423 |
| 4 | Prevention | 804106 |
| 5 | Risk reduction | 19232 |
| 6 | S4 OR S5 | 814132 |
| 7 | (MH "Australia+") OR "Australia" OR (MH "Western Australia") OR (MH "South Australia") OR (MH "Victoria") OR (MH "Tasmania") OR (MH "Queensland") OR (MH "Northern Territory") OR (MH "New South Wales") | 151101 |
| 8 | Protocol | 157276 |
| 9 | (MH "Prospective Studies") OR "cohort profile" | 541724 |
| 10 | (MH "Cross Sectional Studies") OR "cross sectional study" OR (MH "Controlled Before-After Studies") | 300279 |
| 11 | S8 OR S9 OR S10 | 946372 |
| 12 | S3 AND S6 AND S7 AND S11 | 77 |
| 13 | Custom date set to 31/10/2023 - 11/02/2025 | 6 |
