# Supplementary material for: Diversity and inclusivity in Australian dementia prevention research: A mixed methods review
Source: Alzheimers Dement (N Y). 2026 Jul 18;12(3):e70296. doi: 10.1002/trc2.70296 (PMC13380669; doi:10.1002/trc2.70296)
Supplement: Supplementary file 3 — Supporting Information [file TRC2-12-e70296-s007.docx]

**Supplementary File 3**

Data synthesis approach for submitted participant-level data

| **Study name** | **Age** | **Gender** | **Sex** | **Education^a^** | **Geographical Location^b^** | **Birth country** | **Ethnicity** | **Aboriginal and Torres Strait Islander status** | **First language** | **Preferred language** | **SES** | **Relationship status^c^** | **Other** |
| --- | --- | --- | --- | --- | --- | --- | --- | --- | --- | --- | --- | --- | --- |
| **ISLAND [1]** | Age listed | (male/female) | NA | Highest education level: Certificate or Apprenticeship (including Cert 2, 3 or 4), Higher University degree (Honours, Graduate Diploma, Masters or PhD), Other, High School, Diploma / Associate Degree, Bachelor's Degree, Primary School | Postcode listed | Listed | NA | Yes  /No | NA | NA | IRSAD decile | NA | Ancestry |
| **CLS [2]** | Age listed | (male/female) | NA | Total years of education | NA | Listed | NA | NA | NA | NA | NA | NA | NA |
| **MYN [3]** | Age listed (at screening) | (male/female) | NA | Education level: left formal education before age 16, left formal education at age 16, left formal education at age 18, undergraduate degree, Master's degree/post-graduate diploma, PhD | Postcode listed | NA | NA | NA | NA | NA | NA | NA | NA |
| **PISA [4]** | Age listed | (male/female) | NA | Highest level of school completed (Did not go to school, Below year 6, Year 6, Year 7, Year 8, Year 9, Year 10, Year 11, Year 12); Other qualifications completed (Trade qualification, TAFE/VET certificate I or II, TAFE/VET certificate III or IV, TAFE/VET diploma or advanced diploma, Bachelor degree, Bachelor degree with Honours, Graduate diploma or graduate certificate, Masters, Doctorate PhD); Total years of education | Postcode listed | Country listed | Caucasian, Asian, Indigenous Australian/Torres Strait Islander, Pacific Islander, other (listed) | Yes  /No | NA | NA | Main occupation (categorised into manager, professional, technician/trades worker, community/personal service worker, clerical/administrative worker, sales worker, machinery operator/driver, labourer, other); Occupation title; Approximate weekly/annual personal income (categorised) | Relationship status: Single, Married, Living with partner (de facto), Separated, Divorced, Widowed | Birth suburb (if in Australia); Age and year moved to Australia (if born elsewhere); Residence type; Living arrangements ; Country and town/city/suburb in which schooling was completed; Member of veteran or defence forces (yes/no) |
| **LEISURE [5]** | Age listed | (male/female) | NA | Total years of education and Education level (CANTAB level of education): left formal education before age 16, left formal education at age 16, left formal education at age 17-18, undergraduate degree or equivalent, masters degree or equivalent, PhD or equivalent | NA | Australia (1); Asia, Middle East, North Africa, Southern Europe (2); other (3) | NA | Yes  /No | Language listed | NA | NA | NA | NA |
| **MedWalk [6]** | Age listed (at baseline) | (male/female) | NA | Total years of education | State (SA or VIC) | Country listed | Caucasian, Asian, Aboriginal/Torres Strait Islander, Other | Listed under ethnicity variable | NA | Main language spoken at home | NA | NA | Years moved to Australia (if born elsewhere) |
| **ACTIVate [7]** | Age listed (at baseline) | NA | (male/female) | Total years of education | Postcode listed | Country listed | NA | NA | NA | NA | NA | Single, De-facto, Married, Divorced, Widowed | NA |
| **OATS [8]** | Age listed (wave 1) | NA | (male/female) | Total years of education (wave 1) | Location of testing | Country listed | Caucasian, Asian, Indigenous Australian/Torres Strait Islander, African, Pacific Islander, Mixed, Other, N/A, Don't Know | Listed under race (ethnicity) variable | Listed as primary language | Preferred language | Living arrangements (in the community alone; in the community with spouse; in the community with other; in a hostel villa; in a retirement home; other), and Main occupation: manager/admin; professional; assoc professional; tradesperson; advanced clerical/service; intermediate clerical/sales/service; intermediate prod/transport; elementary clerical/sales/service; labourers & related; home duties; other | Married/defacto, separated, divorced, widowed, never married, other | Difficulty learning, writing or reading at school; handedness; handedness; year of retirement; number of children, are you the primary carer for someone (and how long for); pets; mobility aids |
| **MAS [9]** | Age listed (wave 1) | NA | (male/female) | Total years of education and highest qualification (completed tertiary; incomplete tertiary; complete high school and certificate/diploma; incomplete high school and certificate/diplome; completed high school; incomplete high school; primary school) | NA | Country listed | Caucasian, Asian, Indigenous Australian/Torres Strait Islander, Pacific Islander, African, mixed other (listed) | Listed under race (ethnicity) variable | Listed as primary language | Preferred language | Living arrangements (in the community alone; in the community with spouse; in the community with other; in a hostel villa; in a retirement home; other), and Main occupation: manager/admin; professional; assoc professional; tradesperson; advanced clerical/service; intermediate clerical/sales/service; intermediate prod/transport; elementary clerical/sales/service; labourers & related; home duties; other | Married/defacto, divorced, separated, widowed, never married, other | Difficulty learning, writing or reading at school; handedness; year of retirement; number of children, are you the primary carer for someone (and how long for); pets; mobility aids; history of chronic condition/major illnesses; communicating in English; vision |
| **SCS [10]** | Age listed (wave 1) | NA | (male/female) | Total years of education and highest qualification (Incomplete primary school; completed primary school; intermediate certificate; leaving certificate; diploma; degree; postgraduate degree; "777"; missing) | NA | NA | NA | NA | NA | NA | Main occupation (free text) | married or defacto, single and never married, separated, divorced, widowed, other | NA |
| **PATH [11]** | Age listed (wave 1) | (male/female) | NA | Two questions asked: highest level of schooling completed (some primary; all of primary; some of secondary; three/four years of secondary; five/six years of secondary); and highest level of post secondary/tertiary education completed (trade certificate/apprenticeship; technician’s certificate/advanced certificate; certificate other than above; associate diploma; undergraduate diploma; Bachelor’s degree; post graduate diploma/certificate; higher degree; none of the above). | NA | NA | NA | NA | NA | NA | NA | NA | NA |
| **KGOWS [12]** | Age listed | Man/woman | NA | Education level (<10 years, ≥10 years) | Modified Monash Model remoteness classification (1-7) | Australia or ‘Other’ | NA | (All participants identified as Aboriginal or Torres Strait Islander in this study) | English or ‘Other’ | NA | Main lifetime job (skilled/ABS standard occupation category 1-7; or unskilled/ABS standard occupation category 8) | Married/de facto, widowed/divorced, or never married | NA |
| **RESCUE [13]** | Age listed | Male/female | NA | Highest qualification completed (higher school or leaving certificate; school or intermediate certificate; no formal qualifications; certificate/diploma (e.g., childcare, technician); university degree; higher university degree (e.g., grad. Dip., Masters, PhD) | NA | Free text | Oceanian -Australian, South-east Asian, North-west European, Southern and Central Asian (others listed but not selected) | NA | NA | “Do you usually speak a language other than English at home?” | Living arrangements (with partner/spouse; alone; with child/children); main profession (administrative assistant; professional; sales and personal service worker; manager or administrator; para professional; trade; other); income (“how do you manage on the income you have available?) | Married/partnered, widowed/widower, never married, separated, divorced. | NA |
| **OPTIMISE Your Health [14]** | Age listed | (male/female) | NA | Highest qualification completed (did not finish high school i.e., Year 12/Form 6/HSC, completed high school i.e., Year 12/Form 6/HSC, Completed additional higher education i.e., TAFE/trade certificate/diploma/University etc. | NA | Australia, England, New Zealand, India, Italy, Vietnam, Philippines, or ‘other’ (with free text option). | NA | NA | NA | NA | Household type (married or de facto couple only, person living alone, one person living with children, shared household, or other). | NA | Ancestry (English, Irish, Scottish, Italian, German, Chinese, Australian, and/or ‘other (with free text option)). |

*Note.* ISLAND = The Island Study Linking Ageing and Neurodegenerative Disease; CLS = Canberra Longitudinal Study; MYN = Mind Your Nose; PISA = Prospective Imaging Study of Ageing; OATS = Older Australian Twins Study; MAS = Sydney Memory and Ageing Study; SCS = Sydney Centenarian Study; PATH = Personality and Total Health Through Life; KGOWS = Koori Growing Old Well Study; SES = socioeconomic status; IRSAD = Index of Relative Social Advantage and Disadvantage; NA = not applicable (i.e., demographic characteristic not included in submitted dataset).

^a^ Education level data were re-levelled into the following categories for synthesis: high school or lower; certificate, associate degree, diploma or trade; Bachelor’s degree; higher University degree (Masters, post-graduate diploma, or PhD); other.

^b^ Postcode data were used to classify remoteness using the following categories (Modified Monash Model): Metropolitan area; regional centre; large rural town; medium rural town; small rural town; remote community; very remote community.

^c^ Relationship status data were re-levelled into the following categories for synthesis: partnered (living with partner, married, or de-facto); divorced/separated; widowed; never married/single; other.

**References**

[1] Bartlett L, Doherty K, Farrow M, Kim S, Hill E, King A, et al. Island study linking aging and neurodegenerative disease (ISLAND) targeting dementia risk reduction: protocol for a prospective web-based cohort study. JMIR research protocols. 2022;11:e34688.

[2] Christensen H, Mackinnon A, Jorm AF, Korten A, Jacomb P, Hofer SM, et al. The Canberra Longitudinal Study: Design, Aims, Methodology, Outcomes and Recent Empirical Investigations. Aging, Neuropsychology, and Cognition. 2004;11:169-95.

[3] Burke IJM, Chesser C, Brown CPK, Watkins R, Butterworth P, Olofsson JK, et al. Mind your nose: A randomized controlled trial of olfactory-based memory training for older people with subjective cognitive decline. Alzheimer's & Dementia: Translational Research & Clinical Interventions. 2025;11:e70120.

[4] Lupton MK, Robinson GA, Adam RJ, Rose S, Byrne GJ, Salvado O, et al. A prospective cohort study of prodromal Alzheimer's disease: Prospective Imaging Study of Ageing: Genes, Brain and Behaviour (PISA). Neuroimage Clin. 2021;29:102527.

[5] Treacy C, Levenstein JM, Jefferies A, Metse AP, Schaumberg MA, Villani A, et al. The LEISURE Study: A Longitudinal Randomized Controlled Trial Protocol for a Multi-Modal Lifestyle Intervention Study to Reduce Dementia Risk in Healthy Older Adults. Journal of Alzheimer’s Disease. 2023;94:841-56.

[6] Pipingas A, Murphy KJ, Davis CR, Itsiopoulos C, Kingsley M, Scholey A, et al. A Mediterranean Diet and Walking Intervention to Reduce Cognitive Decline and Dementia Risk in Independently Living Older Australians: The MedWalk Randomized Controlled Trial Experimental Protocol, Including COVID-19 Related Modifications and Baseline Characteristics. Journal of Alzheimer’s Disease. 2023;96:409-27.

[7] Smith AE, Wade AT, Olds T, Dumuid D, Breakspear MJ, Laver K, et al. Characterising activity and diet compositions for dementia prevention: protocol for the ACTIVate prospective longitudinal cohort study. BMJ Open. 2022;12:e047888.

[8] Sachdev PS, Lammel A, Trollor JN, Lee T, Wright MJ, Ames D, et al. A Comprehensive Neuropsychiatric Study of Elderly Twins: The Older Australian Twins Study. Twin Research and Human Genetics. 2009;12:573-82.

[9] Sachdev PS, Brodaty H, Reppermund S, Kochan NA, Trollor JN, Draper B, et al. The Sydney Memory and Ageing Study (MAS): methodology and baseline medical and neuropsychiatric characteristics of an elderly epidemiological non-demented cohort of Australians aged 70–90 years. International Psychogeriatrics. 2010;22:1248-64.

[10] Sachdev PS, Levitan C, Crawford J, Sidhu M, Slavin M, Richmond R, et al. The Sydney Centenarian Study: methodology and profile of centenarians and near-centenarians. International Psychogeriatrics. 2013;25:993-1005.

[11] Anstey KJ, Christensen H, Butterworth P, Easteal S, Mackinnon A, Jacomb T, et al. Cohort Profile: The PATH through life project. International Journal of Epidemiology. 2012;41:951-60.

[12] Radford K, Mack HA, Robertson H, Draper B, Chalkley S, Daylight G, et al. The Koori Growing Old Well Study: investigating aging and dementia in urban Aboriginal Australians. International Psychogeriatrics. 2014;26:1033-43.

[13] Gardiner PA. A randomised controlled trial into the effect of reducing prolonged sitting on cognitive function in insufficiently active frail older adults. Australia New Zealand Clinical Trials Registry2017.

[14] Brakenridge CJ, Gardiner PA, Grigg RV, Winkler EAH, Fjeldsoe BS, Schaumberg MA, et al. Sitting less and moving more for improved metabolic and brain health in type 2 diabetes: ‘OPTIMISE your health’ trial protocol. BMC Public Health. 2022;22:929.
