# Supplementary material for: Diversity and inclusivity in Australian dementia prevention research: A mixed methods review
Source: Alzheimers Dement (N Y). 2026 Jul 18;12(3):e70296. doi: 10.1002/trc2.70296 (PMC13380669; doi:10.1002/trc2.70296)

TASK 1: MIRO CHECK

4 minutes

Select an emoji that represents you today and drag it onto your name

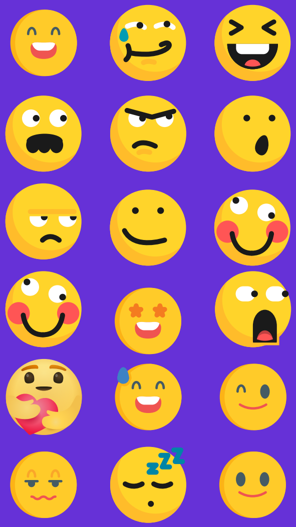

Name 1

Name 2

Name 3

Name 4

Name 5

Name 6

TASK 2: WHY? . . .

20 minutes

As a group, discuss the data on the shiny-app, and capture your thoughts on the prompts below

Why are we collecting the data we are?

Collecting

Why are we publishing the data we are?

Publishing

Why don't we collect other types of demographic data?

Not collecting

Why don't we publish other types of demographic data?

Not publishing

. . . SO WHAT?

Thinking about the information above, consider why it matters (or doesn't matter). Use the prompts below to expand and diversify your discussion (or ignore them completely!)

S	T	E	E	P	L	E	D
Social	Technological	Ecological	Economic	Political	Legal	Ethical	Demographic
Social			Economic	Political	Legal	Ethical	

TASK 3: EXTRAPOLATE

15 minutes

What could be the longer term consequences and opportunities of change?

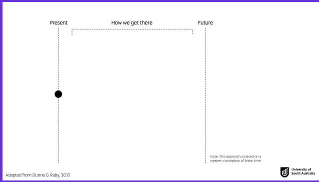

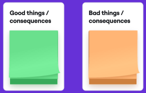

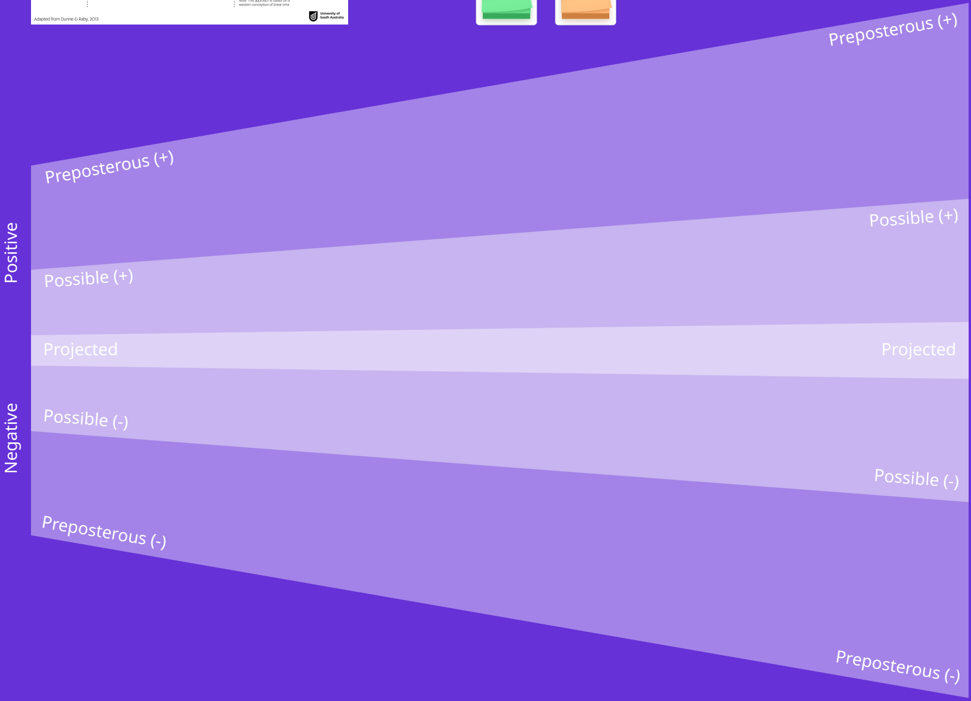

Short term
3 years

Long term
30 years

TASK 4: WHAT CAN . . . DO?

20 minutes

Part 1: Add ideas for initiatives / policies / SOPs to address current and / or future challenges you have identified

GOVERNMENTS

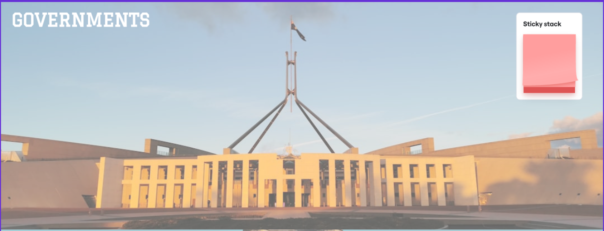

INSTITUTIONS / SOCIETIES / ORGANISATIONS

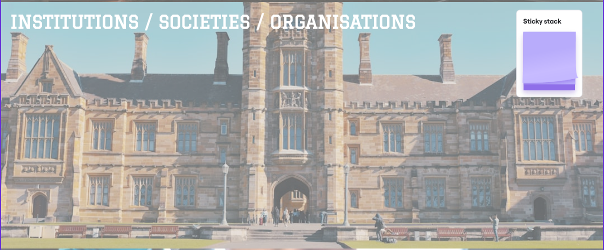

INDIVIDUAL RESEARCHERS

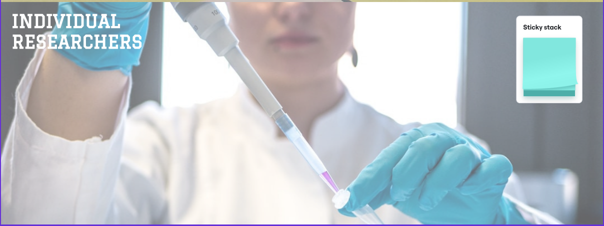

Part 2: Drag the ideas anybody in the group thinks could be taken forward into policy recommendations into the space below.

TASK 5: POLICY RECOMMENDATIONS

20 minutes

Assemble preferred policy recommendations from all groups in the space below. Each group will be asked briefly describe the ideas they have added. We will then provide an opportunity to review these according to the Gradients of Agreement below with a particular focus on levels 1, 2, 3, 4 and 8.

GOVERNMENT

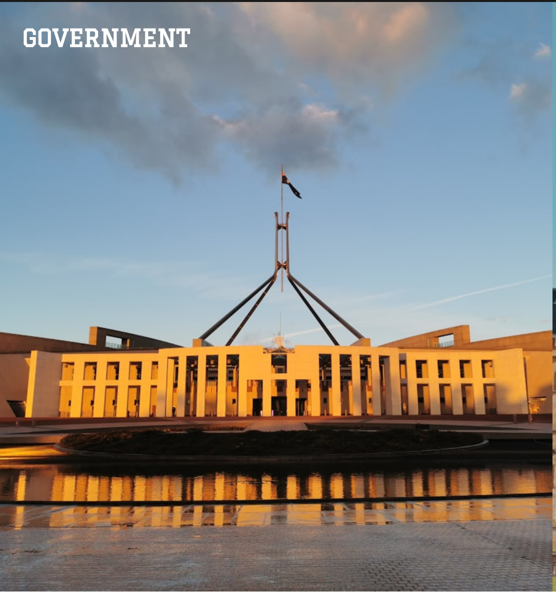

INSTITUTIONS / SOCIETIES / ORGANISATIONS

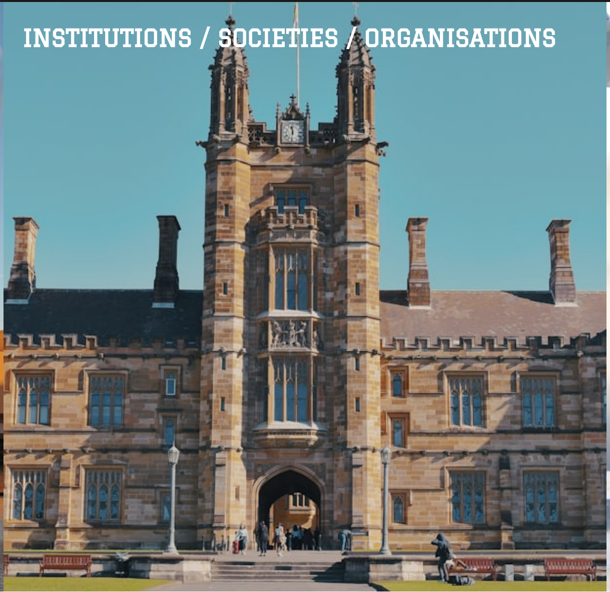

INDIVIDUAL RESEARCHERS

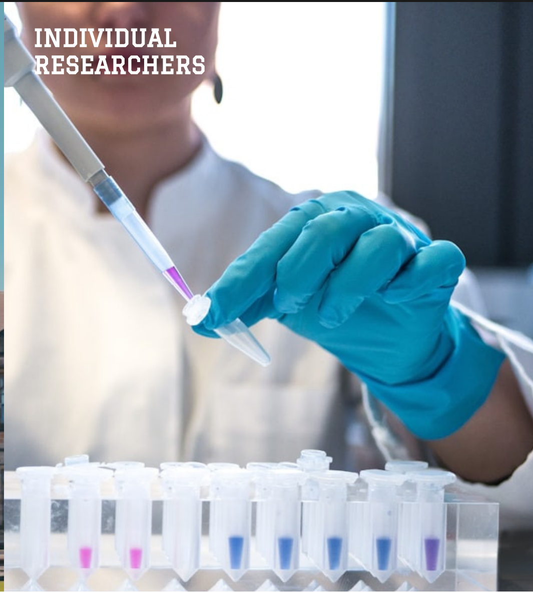

Supplement: Supplementary file 4 — Supporting Information [file TRC2-12-e70296-s008.pdf]
