# Supplementary material for: Diversity and inclusivity in Australian dementia prevention research: A mixed methods review
Source: Alzheimers Dement (N Y). 2026 Jul 18;12(3):e70296. doi: 10.1002/trc2.70296 (PMC13380669; doi:10.1002/trc2.70296)
Supplement: Supplementary file 5 — Supporting Information [file TRC2-12-e70296-s009.pdf]

Thank you for your involvement in the Dementia mapping study so far!

This study aims to summarise the characteristics of participants (e.g., age, sex, gender, ethnicity, geographical location, sexual orientation) involved in dementia prevention research using both published cohort-level data and participant-level data for Australian cohort studies and randomised controlled trials with available demographic data. To achieve this aim, a systematic literature search was conducted, and authors (identified through the literature search, a grant search and a search of clinical trials registries) were contacted to request de-identified participant-level demographic data.

An additional aim of this study was to present the findings in a workshop with Australian dementia researchers, and as a result of your participation in this workshop last month, we have formulated a list of recommendations for governments, institutions/societies and individual researchers.

In this survey will be 3 sets of policy recommendations that you will be asked questions about. In each of the sections (government, institutions/societies and individual

researchers), you will be asked to rank the recommendations by their importance, and to indicate your level of agreement for these recommendations. This ranking will determine the order of these recommendations in the manuscript.

Please get in contact if you have any further questions: ashleigh.smith@unisa.edu.au

Government Recommendations

Recommendations for **Government**

Please rank government recommendations by importance (1 being most important)

Funding bodies should mandate sample representativeness and prioritise reserch being conducted with diverse communities: Including having lived-experience involvement in each stage of research

Ensure there is a focus on diversity and social determinants of health in medical and scientific research

Policies related to dementia prevention and risk reduction must be developed through engagement with the target population and must live beyond the political term

Funding bodies should develop specific grant rounds focussed on diversity in dementia research and follow up on sample representativeness: specifically focussing on population representative

cohort studies and taking into account factors such as the track record of diversity in the previous samples of the CI

Focus on systemic change to allow individuals from underrepresneted communities to engage in research: teaching the importance of diversity in schools, providing specific opportunities for diverse groups to engage in research (including access to university)

Have a greater representation of diverse populations on health advisory boards

Develop policies which protect data collection to facilitate collection of rich demographic data

Have a minimum list of demographics which should be collected in government-funded dementia risk reduction research

Identify which groups are missing from census data and national level data sets

Eliminate barriers diverse groups face when volunteering for research

Dedicate funding to building relationships with diverse communities, both prior to the start of the research and during the project development phase

Develop policies in conjunction with researchers through systematic reviews and meta-analyses

Support data linkage and communication between governments, research institutions and researchers: make this process easier practically and financially

Have more centralised support for researchers to develop diversity measures into their research

We are going to ask you to review the policy options you have just ranked again, to consider how strongly you feel about each of them. Please review the gradients of agreement framework below, then consider if any of the policy options fall into the “1: Veto” or “8: Whole Heartedly Endorse” categories.

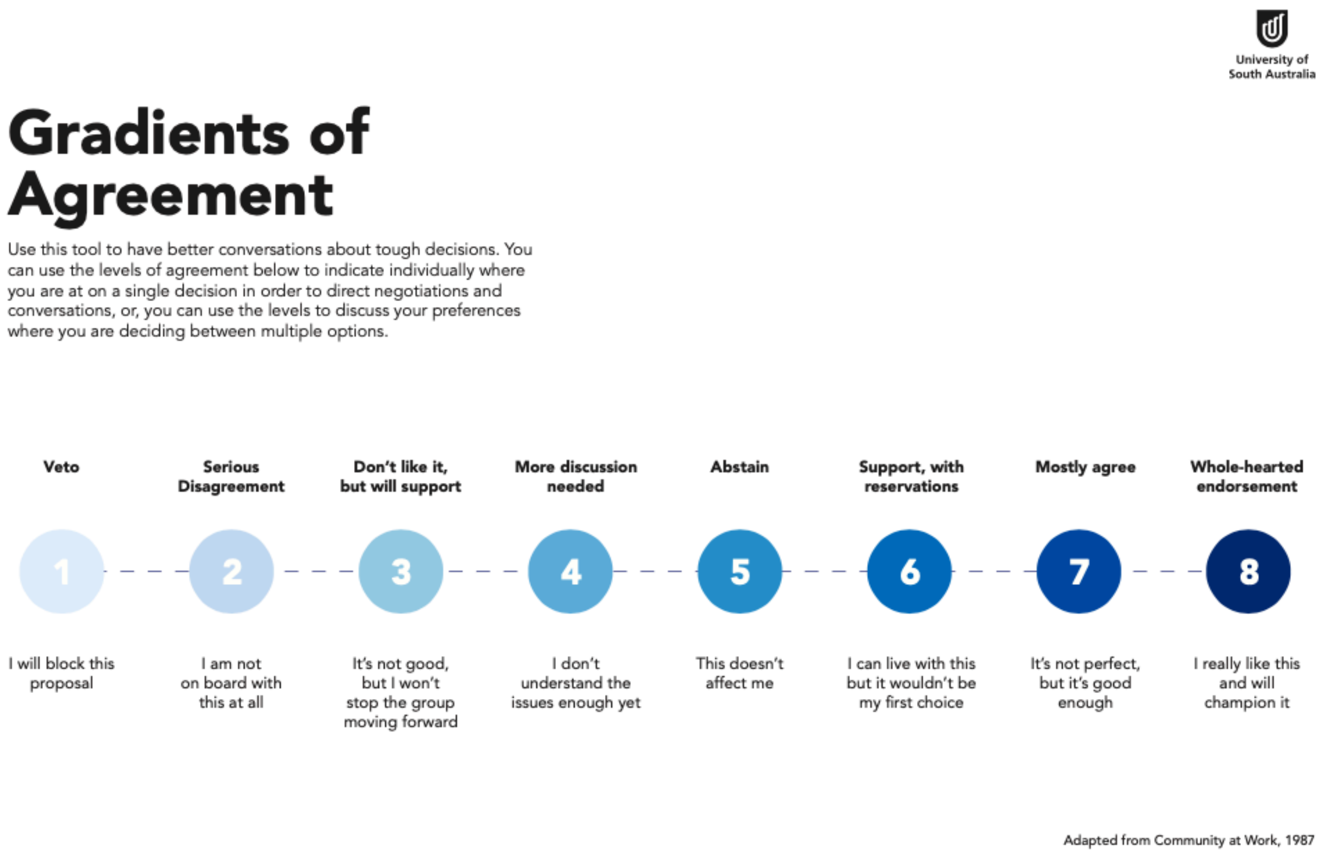

Please identify any options that would fit into each extreme of the gradients of agreement framework
(Please note, we are only capturing the ratings on each extreme of the scale, therefore if your rating is from 2 - 7, the row should be left blank with neither options selected)

	Level 1: Veto	Level 8: Whole-heartedly endorse
Funding bodies should mandate sample representativeness and prioritise reserch being conducted with diverse communities: Including having lived-experience involvement in each stage of research	☐	☐
Ensure there is a focus on diversity and social determinants of health in medical and scientific research	☐	☐
Policies related to dementia prevention and risk reduction must be developed through engagement with the target population and must live beyond the political term	☐	☐
Funding bodies should develop specific grant rounds focussed on diversity in dementia research and follow up on sample representativeness: specifically focussing on population representative cohort studies and taking into account factors such as the track record of diversity in the previous samples of the CI	☐	☐

	Level 1: Veto	Level 8: Whole-heartedly endorse
Focus on systemic change to allow individuals from underrepresneted communities to engage in research: teaching the importance of diversity in schools, providing specific opportunities for diverse groups to engage in research (including access to university)	☐	☐
Have a greater representation of diverse populations on health advisory boards	☐	☐
Develop policies which protect data collection to facilitate collection of rich demographic data	☐	☐
Have a minimum list of demographics which should be collected in government-funded dementia risk reduction research	☐	☐
Identify which groups are missing from census data and national level data sets	☐	☐
Eliminate barriers diverse groups face when volunteering for research	☐	☐

	Level 1: Veto	Level 8: Whole-heartedly endorse
Dedicate funding to building relationships with diverse communities, both prior to the start of the research and during the project development phase	☐	☐
Develop policies in conjunction with researchers through systematic reviews and meta-analyses	☐	☐
Support data linkage and communication between governments, research institutions and researchers: make this process easier practically and financially	☐	☐
Have more centralised support for researchers to develop diversity measures into their research	☐	☐

If you have chosen Level 1: Veto for any of the recommendations in the previous question, please explain why

Recommendations for **Institutions/Societies**

Please rank institutions/societies recommendations by importance (1 being most important)

Institutions and journals should develop reporting guidelines for diversity in research: making it the gold standard to collect a broad range of demographic data, and developing standardised terms for improved harmonisation of data (both within Australia and internationally)

Improve diversity awareness and training for researchers: including asking CIs to complete implicit bias testing, and having this training implemented much earlier in their career (in undergraduate)

Increase support to encourage retention of researchers from diverse communities

Institutions should form better recruitment policies and procedures: including reviewing these policies to identify barriers to diverse recruitment

Facilitate opportunities for collaboration with communities and between research from a diverse range of backgrounds

Develop committees and teams dedicated to diversity: where representatives are appropriately re-imbursed for their time and expertise: this committee could assist with employing researchers with lived experience to collect data

Mandate co-design of research and grants

Increase the ease of data-sharing agreements between institutions

Emphasise the holistic, life-course conceptualisation of dementia: including a greater integration on social models of health

Journals should increase support for opinion pieces and commentaries encouraging discourse, and for studied which highlight systemic issues to the government

Continue to fund studies beyond their original terms as a mechanism to continue legacy cohort studies

Ensure there is sufficient funding for meaningful investigation into sub-group analyses

Ethics applications should have a bigger focus on diversity as an ethical issue: increase check-ins along the way and be more flexible around different consent procedures for inclusion of underrepresented populations

Encourage open access data frameworks/policies from the beginning

Make efforts to understand different procedures and knowledge which look beyond 'Western' paradigms

We are going to ask you to review the policy options you have just ranked again, to consider how strongly you feel about each of them. Please review the gradients of agreement framework below, then consider if any of the policy options fall into the “1: Veto” or “8: Whole Heartedly Endorse” categories.

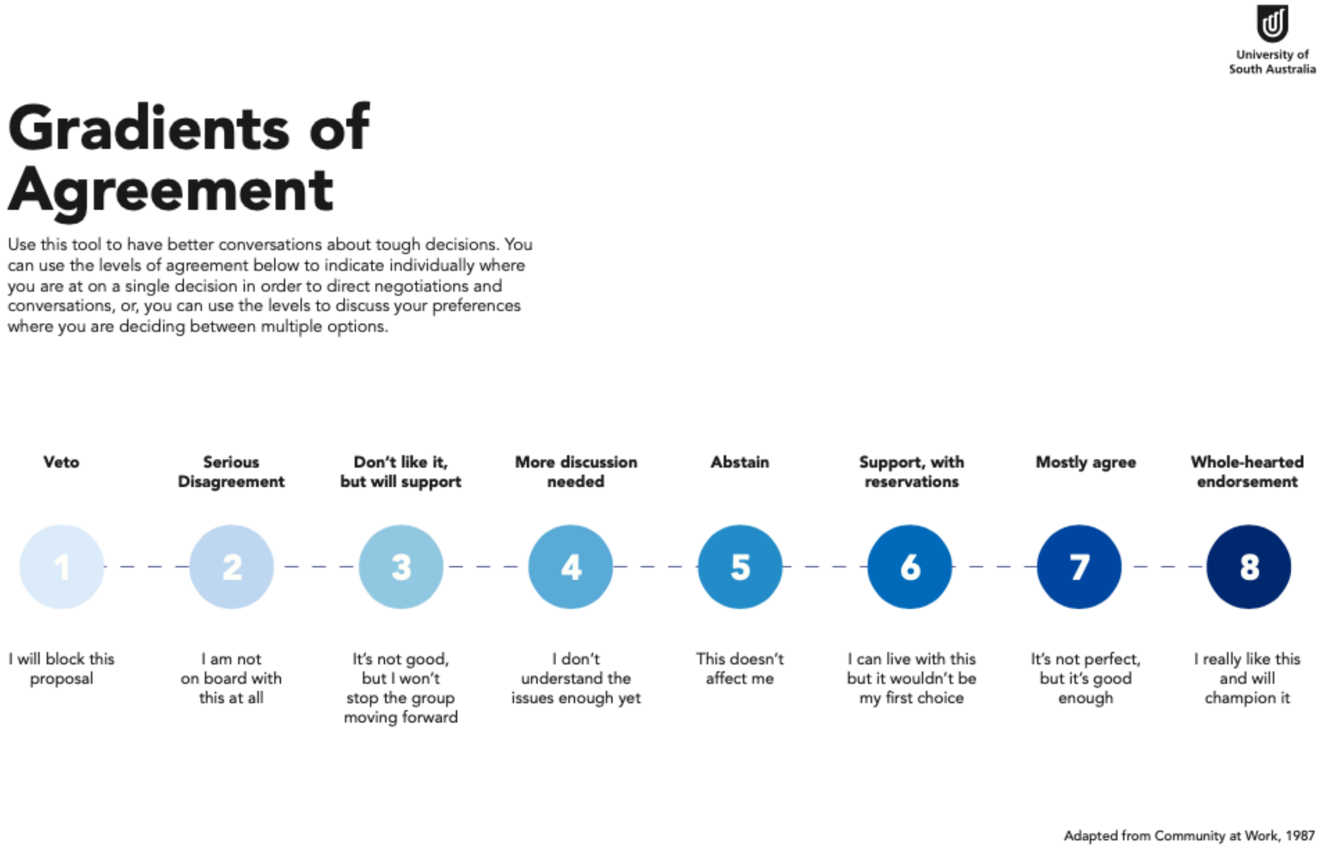

Please identify any options that would fit into each extreme of the gradients of agreement framework
(Please note, we are only capturing the ratings on each extreme of the scale, therefore if your rating is from 2 - 7, the row should be left blank with neither options selected)

	Level 1: Veto	Level 8: Whole-heartedly endorse
Institutions and journals should develop reporting guidelines for diversity in research: making it the gold standard to collect a broad range of demographic data, and developing standardised terms for improved harmonisation of data (both within Australia and internationally)	☐	☐
Improve diversity awareness and training for researchers: including asking CIs to complete implicit bias testing, and having this training implemented much earlier in their career (in undergraduate)	☐	☐
Increase support to encourage retention of researchers from diverse communities	☐	☐
Institutions should form better recruitment policies and procedures: including reviewing these policies to identify barriers to diverse recruitment	☐	☐
Facilitate opportunities for collaboration with communities and between research from a diverse range of backgrounds	☐	☐

	Level 1: Veto	Level 8: Whole-heartedly endorse
Develop committees and teams dedicated to diversity: where representatives are appropriately re-imbursed for their time and expertise: this committee could assist with employing researchers with lived experience to collect data	☐	☐
Mandate co-design of research and grants	☐	☐
Increase the ease of data-sharing agreements between institutions	☐	☐
Emphasise the holistic, life-course conceptualisation of dementia: including a greater integration on social models of health	☐	☐
Journals should increase support for opinion pieces and commentaries encouraging discourse, and for studied which highlight systemic issues to the government	☐	☐
Continue to fund studies beyond their original terms as a mechanism to continue legacy cohort studies	☐	☐

	Level 1: Veto	Level 8: Whole-heartedly endorse
Ensure there is sufficient funding for meaningful investigation into sub-group analyses	☐	☐
Ethics applications should have a bigger focus on diversity as an ethical issue: increase check-ins along the way and be more flexible around different consent procedures for inclusion of underrepresented populations	☐	☐
Encourage open access data frameworks/policies from the beginning	☐	☐
Make efforts to understand different procedures and knowledge which look beyond 'Western' paradigms	☐	☐

If you have chosen Level 1: Veto for any of the recommendations in the previous question, please explain why

Recommendations for **Individual Researchers**

Please rank individual researcher recommendations by importance (1 being most important)

Assess and address personal and positional biases in your knowledge and experiences: engage in the necessary trainings to address these

Make efforts to develop connections with the communities you are researching

Budget and resource projects properly to facilitate inclusion: including paying researchers for their time and expertise

Collaborate with researchers who have expertise in researching diverse populations, those with lived experience and researchers beyond the dementia bubble: increase co-design practices, build in diversity from the beginning of the project and not as an afterthought

Be transparent in communicating the diversity of your sample

Carefully consider the tools being used to assess your sample: choose tools which are inclusive

Be intentional about recruitment methodologies

Keep up with and help to pioneer the gold standard of demographic data collecting and reporting

Support/mentor diverse students

Work to support a change in mindset for diversity to be at the forefront of all research

Use the data you already have to investigate samples and empirically demonstrate the power of representative samples: including collaboration on cohort studies

We are going to ask you to review the policy options you have just ranked again, to consider how strongly you feel about each of them. Please review the gradients of agreement framework below, then consider if any of the policy options fall into the "1: Veto" or "8: Whole Heartedly Endorse" categories.

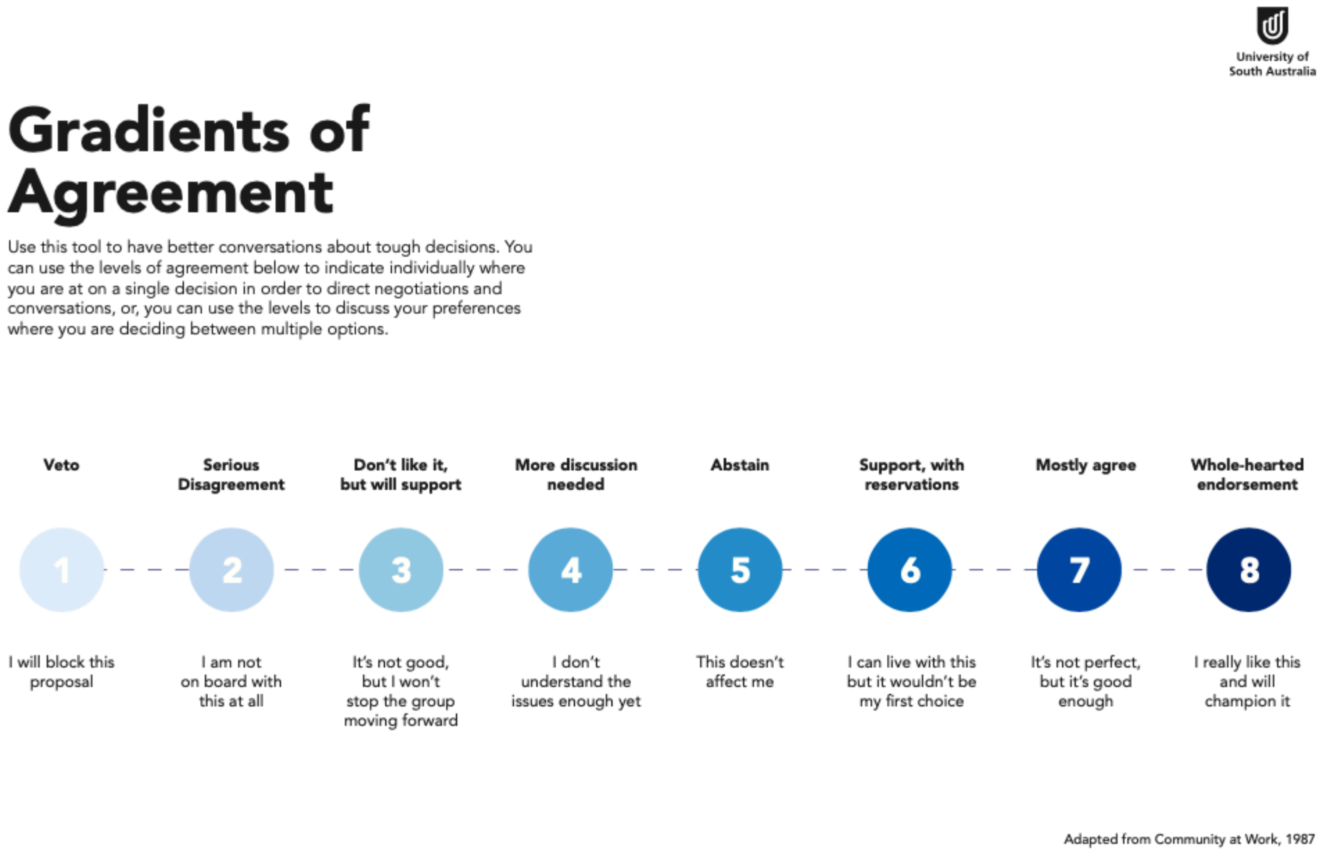

Please identify any options that would fit into each extreme of the gradients of agreement framework (Please note, we are only capturing the ratings on each extreme of the scale, therefore if your rating is from 2 - 7, the row should be left blank with neither options selected)

	Level 1: Veto	Level 8: Whole-heartedly endorse
Assess and address personal and positional biases in your knowledge and experiences: engage in the necessary trainings to address these	☐	☐
Make efforts to develop connections with the communities you are researching	☐	☐
Budget and resource projects properly to facilitate inclusion: including paying researchers for their time and expertise	☐	☐
Collaborate with researchers who have expertise in researching diverse populations, those with lived experience and researchers beyond the dementia bubble: increase co-design practices, build in diversity from the beginning of the project and not as an afterthought	☐	☐
Be transparent in communicating the diversity of your sample	☐	☐

	Level 1: Veto	Level 8: Whole-heartedly endorse
Carefully consider the tools being used to assess your sample: choose tools which are inclusive	☐	☐
Be intentional about recruitment methodologies	☐	☐
Keep up with and help to pioneer the gold standard of demographic data collecting and reporting	☐	☐
Support/mentor diverse students	☐	☐
Work to support a change in mindset for diversity to be at the forefront of all research	☐	☐
Use the data you already have to investigate samples and empirically demonstrate the power of representative samples: including collaboration on cohort studies	☐	☐

If you have chosen Level 1: Veto for any of the recommendations in the previous question, please explain why

Please detail any additional feedback or comments for the authors

Powered by Qualtrics
