# Supplementary figures and images for: Diversity and inclusivity in Australian dementia prevention research: A mixed methods review
Source: Alzheimers Dement (N Y). 2026 Jul 18;12(3):e70296. doi: 10.1002/trc2.70296 (PMC13380669; doi:10.1002/trc2.70296)

## Supplementary File 6

### PRISMA flow diagram

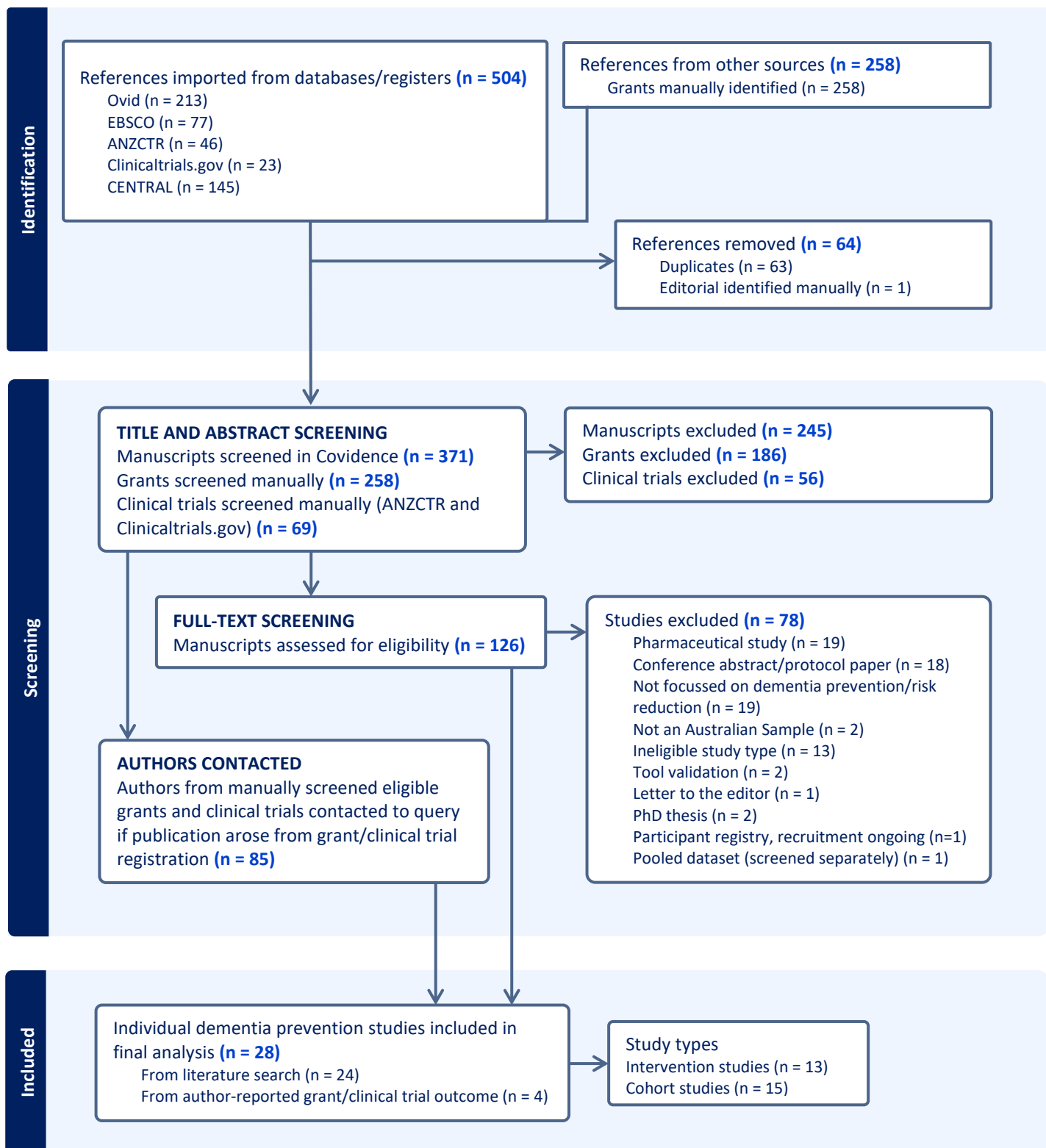

Supplement: Supplementary file 6 — Supporting Information [file TRC2-12-e70296-s003.pdf]
