# Supplementary material for: Diversity and inclusivity in Australian dementia prevention research: A mixed methods review
Source: Alzheimers Dement (N Y). 2026 Jul 18;12(3):e70296. doi: 10.1002/trc2.70296 (PMC13380669; doi:10.1002/trc2.70296)
Supplement: Supplementary file 7 — Supporting Information [file TRC2-12-e70296-s005.docx]

**Supplementary File 7**

List of published dementia prevention cohort and intervention studies included in data synthesis (n=28)

| **Study name** | **Study design** | **Year of study initiation** | **Year of affiliated publication** | **Sample size** | **Also included in participant-level data analysis** |
| --- | --- | --- | --- | --- | --- |
| Canberra Longitudinal Study (CLS) | Cohort | 1990 | 2011 | 687 | Y |
| Women's Healthy Ageing Project (WHAP) | Cohort | 1991 | 1994 | 1503 | - |
| The Australian Longitudinal Study of Ageing (ALSA) | Cohort | 1992 | 2016 | 2087 | - |
| Melbourne Longitudinal Studied on Healthy Ageing (MELSHA) | Cohort | 1994 | 2010 | 1000 | - |
| Personality and Total Health Through Life (PATH) | Cohort | 2001 | 2021 | 7485 | Y |
| Older Australian Twin Study (OATS) | Cohort | 2006 | 2013 | 623 | Y |
| Sydney Memory and Ageing Study (MAS) | Cohort | 2007 | 2010 | 1037 | Y |
| Hunter Community Study | Cohort | 2007 | 2010 | 3207 | - |
| Australian Imaging Biomarkers and Lifestyle Flagship Study of Aging (AIBL) | Cohort | 2008 | 2009 | 768 | - |
| Fish Oil to Prevent Cognitive Decline | Intervention | 2008 | 2018 | 391 | - |
| Curcumin and Cognition | Intervention | 2011 | 2016 | 96 | - |
| Koori Growing Old Well Study (KGOWS) | Cohort | 2012 | 2015 | 336 | Y |
| Body Brain Life (BBL) | Intervention | 2013 | 2015 | 176 | - |
| Tasmanian Healthy Brain Project (THBP) | Cohort | 2014 | 2018 | 444 | - |
| Protein Enriched Diet | Intervention | 2015 | 2020 | 154 | - |
| MedDairy trial | Intervention | 2016 | 2018 | 41 | - |
| Body Brain Life - General Practice | Intervention | 2017 | 2020 | 125 | - |
| Protein Omega-3 and Vitamin D Exercise Research (PONDER) study | Intervention | 2017 | 2022 | 147 | - |
| Body Brain Life for Cognitive Decline (BBL-CD) | Intervention | 2017 | 2023 | 119 | - |
| MedPork trial | Intervention | 2017 | 2019 | 33 | - |
| The Island Study Linking Ageing and Neurodegenerative Disease (ISLAND) | Cohort | 2020 | 2022 | 6410 | Y |
| Prospective Imaging Study of Ageing: Genes, Brain and Behaviour (PISA) | Cohort | 2020 | 2021 | 4082 | Y |
| ACTIVate | Cohort | 2020 | 2024 | 417 | Y |
| BRAIN BOOTCAMP | Intervention | 2021 | 2023 | 857 | - |
| MedWalk | Intervention | 2022 | 2023 | 157 | Y |
|  |  |  |  |  |  |
| The Sydney Centenarian Study (SCS) | Cohort | NA | 2019 | 207 | Y |
| Promoting Healthy Ageing with Cognitive Exercise (PACE) study | Intervention | NA | 2015 | 160 | - |
| LEISURE | Intervention | NA | 2024 | 98 | Y |

*Note.* ‘Y’ indicates datasets where de-identified participant-level data were submitted for synthesis in participant-level data analysis, as well as being included in published data analysis. NA = data not available.
