# Supplementary material for: Diversity and inclusivity in Australian dementia prevention research: A mixed methods review
Source: Alzheimers Dement (N Y). 2026 Jul 18;12(3):e70296. doi: 10.1002/trc2.70296 (PMC13380669; doi:10.1002/trc2.70296)
Supplement: Supplementary file 8 — Supporting Information [file TRC2-12-e70296-s010.docx]

**Supplementary File 8**

Variables reported in published studies

| **Study design** | **Study name** | **Year of publication** | **Biological Sex** | **Gender** | **Age** | **Sexual orientation** | **Education** | **Nationality** | **Ethnicity** | **Aboriginal and Torres Strait Islander status** | **Socioeconomic status** | **Marital status** | **Geographical location** | **First or preferred language** | **Country of birth** |
| --- | --- | --- | --- | --- | --- | --- | --- | --- | --- | --- | --- | --- | --- | --- | --- |
| *Cohort* | Women's Healthy Ageing Project (WHAP) | 1994 | **Y** | - | **Y** | - | **Y** | **-** | **-** | **-** | **Y** | **Y** | **Y** | **-** | **-** |
|  | Australian Imaging Biomarkers and Lifestyle Flagship Study of Aging (AIBL) | 2009 | **-** | **Y** | **Y** | **-** | **Y** | **-** | **-** | **-** | **-** | **Y** | **-** | **-** | **-** |
|  | Sydney Memory and Ageing Study (MAS) | 2010 | **Y** | **-** | **Y** | **-** | **Y** | **-** | **-** | **-** | **Y** | **-** | **Y** | **Y** | **Y** |
|  | Melbourne Longitudinal Studies on Healthy Ageing (MELSHA) | 2010 | **-** | **Y** | **Y** | **-** | **Y** | **-** | **-** | **-** | **Y** | **Y** | **Y** | **-** | **-** |
|  | Hunter Community Study | 2010 | **-** | **Y** | **Y** | **-** | **Y** | **-** | **-** | **-** | **Y** | **Y** | **Y** | **-** | **-** |
|  | Canberra Longitudinal Study | 2011 | **-** | **Y** | **Y** | **-** | **Y** | **-** | **-** | **-** | **-** | **Y** | **Y** | **-** | **-** |
|  | Older Australian Twin Study | 2013 | **Y** | **-** | **Y** | **-** | **Y** | **-** | **-** | **-** | **Y** | **-** | **Y** | **Y** | **Y** |
|  | Koori Growing Old Well Study (KGOWS) | 2015 | **Y** | **-** | **Y** | **-** | **Y** | **Y** | **Y** | **Y** | **Y** | **Y** | **Y** | **Y** | **-** |
|  | The Australian Longitudinal Study of Ageing (ALSA) | 2016 | **-** | **Y** | **Y** | **-** | **Y** | **-** | **-** | **-** | **Y** | **Y** | **Y** | **-** | **-** |
|  | Tasmanian Healthy Brain Project (THBP) | 2018 | **Y** | **-** | **Y** | **-** | **Y** | **-** | **-** | **-** | **-** | **-** | **Y** | **-** | **-** |
|  | The Sydney Centenarian Study | 2019 | **Y** | **-** | **Y** | **-** | **Y** | **-** | **Y** | **-** | **Y** | **Y** | **Y** | **Y** | **-** |
|  | Personality and Total Health Study (PATH) | 2021 | **Y** | **-** | **Y** | **-** | **Y** | **-** | **-** | **-** | **Y** | **Y** | **Y** | **-** | **-** |
|  | Prospective Imaging Study of Ageing: Genes, Brain and Behaviour (PISA) | 2021 | **Y** | **-** | **Y** | **-** | **Y** | **-** | **Y** | **Y** | **-** | **-** | **-** | **-** | **-** |
|  | The Island Study Linking Ageing and Neurodegenerative Disease (ISLAND) | 2022 | **-** | **Y** | **Y** | **-** | **Y** | **-** | **-** | **-** | **Y** | **-** | **Y** | **-** | **-** |
|  | BRAIN BOOTCAMP | 2023 | **-** | **Y** | **Y** | **-** | **Y** | **-** | **-** | **-** | **Y** | **-** | **Y** | **-** | **Y** |
|  | ACTIVate | 2024 | **Y** | **-** | **Y** | **-** | **Y** | **-** | **-** | **-** | **-** | **Y** | **Y** | **-** | **Y** |
| *Intervention* | Body Brain Life (BBL) | 2015 | **Y** | **-** | **Y** | **-** | **Y** | **-** | **-** | **-** | **-** | **-** | **Y** | **-** | **-** |
|  | Promoting Healthy Ageing with Cognitive Exercise (PACE) study | 2015 | **-** | **Y** | **Y** | **-** | **Y** | **-** | **-** | **-** | **-** | **-** | **-** | **-** | **-** |
|  | Curcumin and Cognition | 2016 | **Y** | **-** | **Y** | **-** | **Y** | **-** | **-** | **-** | **-** | **-** | **Y** | **-** | **-** |
|  | MedDairy trial | 2018 | **-** | **Y** | **Y** | **-** | **Y** | **-** | **-** | **-** | **-** | **-** | **Y** | **-** | **-** |
|  | Fish Oil to Prevent Cognitive Decline | 2018 | **Y** | **-** | **Y** | **-** | **Y** | **-** | **-** | **-** | **-** | **-** | **Y** | **-** | **-** |
|  | MedPork trial | 2019 | **Y** | **-** | **Y** | **-** | **Y** | **-** | **-** | **-** | **-** | **-** | **-** | **-** | **-** |
|  | Protein Enriched Diet | 2020 | **-** | **Y** | **Y** | **-** | **Y** | **-** | **Y** | **Y** | **-** | **-** | **Y** | **-** | **Y** |
|  | Body Brain Life - General Practice | 2020 | **Y** | **-** | **Y** | **-** | **Y** | **-** | **-** | **-** | **-** | **-** | **Y** | **-** | **-** |
|  | Protein Omega-3 and Vitamin D Exercise Research (PONDER) study | 2022 | **Y** | **-** | **Y** | **-** | **Y** | **-** | **-** | **-** | **Y** | **-** | **Y** | **-** | **-** |
|  | Body Brain Life for Cognitive Decline (BBL-CD) | 2023 | **-** | **Y** | **Y** | **-** | **Y** | **-** | **-** | **-** | **-** | **-** | **Y** | **-** | **-** |
|  | MedWalk | 2023 | **Y** | **-** | **Y** | **-** | **Y** | **-** | **Y** | **-** | **Y** | **Y** | **Y** | **-** | **Y** |
|  | LEISURE | 2024 | **Y** | **-** | **Y** | **-** | **Y** | **-** | **-** | **-** | **-** | **-** | **Y** | **-** | **-** |

*Note.* ‘Y’ indicates that variable was reported in published study; dash (-) indicates that variable was not reported in published study.
