# Supplementary material for: Diversity and inclusivity in Australian dementia prevention research: A mixed methods review
Source: Alzheimers Dement (N Y). 2026 Jul 18;12(3):e70296. doi: 10.1002/trc2.70296 (PMC13380669; doi:10.1002/trc2.70296)
Supplement: Supplementary file 9 — Supporting Information [file TRC2-12-e70296-s001.docx]

**Supplementary File 9**

Recommendations generated through vision workshops

Table 1. Recommendations for government-level change in dementia prevention research practice

| **Rank** | **Recommendations/priorities** | **Condorcet** | **Valency** | **Whole-hearted endorsement by experts (n, %)** |
| --- | --- | --- | --- | --- |
| 1 | Ensure there is a focus on diversity and social determinants of health in medical and scientific research | 14 | 14 | 16 (64%) |
| 2 | Policies related to dementia prevention and risk reduction must be developed through engagement with the target population and must live beyond the political term | 13 | 13 | 18 (72%) |
| 3 | Have a minimum list of demographics which should be collected in government-funded dementia risk reduction research | 12 | 12 | 11 (44%) |
| 4 | Funding bodies should develop specific grant rounds focused on diversity in dementia research and follow up on sample representativeness, specifically focusing on population-representative cohort studies and taking into account factors such as the track record of diversity in the previous samples of the Chief Investigator | 10 | 10 | 11 (44%) |
| 5 | Focus on systemic change to allow individuals from under-represented communities to engage in research: teaching the importance of diversity in schools, providing specific opportunities for diverse groups to engage in research (including access to university) | 9 | 9 | 12 (48%) |
| 6 | Have a greater representation of diverse populations on health advisory boards | 8 | 8 | 13 (52%) |
| 7 | Dedicate funding to building relationships with diverse communities, both prior to the start of the research and during the project development phase | 7 | 7 | 10 (40%) |
| 8 | Eliminate barriers that diverse groups face when volunteering for research | 6 | 6 | 12 (48%) |
| 9 | Support data linkage and communication between governments, research institutions and researchers - make this process easier practically and financially | 5 | 4 | 10 (40%) |
| 10 | Have more centralised support for researchers to develop diversity measures into their research | 5 | 4 | 7 (28%) |
| 11 | Develop policies which protect data collection to facilitate collection of rich demographic data | 5 | 3 | 4 (16%) |
| 11 | Identify which groups are missing from census data and national-level data sets | 5 | 3 | 4 (16%) |
| 12 | Develop policies in conjunction with researchers through systematic reviews and meta-analyses | 1 | 1 | 7 (28%) |

***Note.*** Recommendations for government-level change to improve recruitment and reporting practices in dementia prevention research. Recommendations were generated by Australian dementia researchers and ranked using a multiple criteria decision analysis. ‘Condorcet’ = how many times the recommendation out-ranked all lower-ranked options. ‘Valency’ = how many experts agreed with the position in the rank.

Table 2. Recommendations for institution-level change in dementia prevention research practice

| **Rank** | **Recommendations/priorities** | **Condorcet** | **Valency** | **Whole-hearted endorsement by experts (n, %)** |
| --- | --- | --- | --- | --- |
| 1 | Institutions and journals should develop reporting guidelines for diversity in research, making it the gold standard to collect a broad range of demographic data, and developing standardised terms for improved harmonisation of data (both within Australia and internationally) | 15 | 15 | 15 (60%) |
| 2 | Improve diversity awareness and training for researchers, including asking Chief Investigators to complete implicit bias testing, and having this training implemented much earlier in their career (i.e., in undergraduate years) | 14 | 14 | 10 (40%) |
| 3 | Increase support to encourage retention of researchers from diverse communities | 13 | 13 | 12 (48%) |
| 4 | Institutions should form better recruitment policies and procedures, including reviewing these policies to identify barriers to diverse recruitment | 12 | 12 | 11 (44%) |
| 5 | Develop committees and teams dedicated to diversity, where representatives are appropriately re-imbursed for their time and expertise. This committee could assist with employing researchers with lived experience to collect data | 11 | 11 | 7 (28%) |
| 6 | Facilitate opportunities for collaboration with communities and between research from a diverse range of backgrounds | 10 | 10 | 9 (36%) |
| 7 | Continue to fund studies beyond their original terms as a mechanism to continue legacy cohort studies | 9 | 7 | 11 (44%) |
| 8 | Ethics applications should have a bigger focus on diversity as an ethical issue. Increase check-ins along the way and be more flexible around different consent procedures for inclusion of underrepresented populations | 9 | 7 | 7 (28%) |
| 9 | Increase the ease of data-sharing agreements between institutions | 9 | 6 | 6 (24%) |
| 10 | Emphasise the holistic, life-course conceptualisation of dementia, including a greater integration on social models of health | 9 | 6 | 6 (24%) |
| 11 | Make efforts to understand different procedures and knowledge which look beyond 'Western' paradigms | 9 | 4 | 6 (24%) |
| 12 | Ensure there is sufficient funding for meaningful investigation into sub-group analyses | 9 | 4 | 5 (20%) |
| 13 | Journals should increase support for opinion pieces and commentaries encouraging discourse, and for studies which highlight systemic issues to the government | 2 | 2 | 3 (12%) |
| 14 | Encourage open access data frameworks/policies from the beginning | 1 | 1 | 5 (20%) |

***Note.*** Recommendations for institution-level change to improve recruitment and reporting practices in dementia prevention research. Recommendations were generated by Australian dementia researchers and ranked using a multiple criteria decision analysis. ‘Condorcet’ = how many times the recommendation out-ranked all lower-ranked options. ‘Valency’ = how many experts agreed with the position in the rank.

Table 3. Recommendations for researcher-level change in dementia prevention research practice

| **Rank** | **Recommendations/priorities** | **Condorcet** | **Valency** | **Whole-hearted endorsement by experts (n, %)** |
| --- | --- | --- | --- | --- |
| 1 | Make efforts to develop connections with the communities you are researching | 11 | 11 | 16 (64%) |
| 2 | Assess and address personal and positional biases in your knowledge and experience. Engage in the necessary trainings to address these | 10 | 10 | 13 (52%) |
| 3 | Collaborate with researchers who have expertise in researching diverse populations, those with lived experience and researchers beyond the “dementia bubble”. Increase co-design practices, build in diversity from the beginning of the project and not as an afterthought | 9 | 9 | 15 (60%) |
| 4 | Budget and resource projects properly to facilitate inclusion, including paying researchers for their time and expertise | 8 | 8 | 11 (44%) |
| 5 | Be transparent in communicating the diversity of your sample | 7 | 7 | 10 (40%) |
| 6 | Carefully consider the tools being used to assess your sample. Choose tools which are inclusive | 6 | 6 | 9 (36%) |
| 7 | Be intentional about recruitment methodologies | 5 | 5 | 6 (24%) |
| 8 | Keep up with and help to pioneer the gold standard of demographic data collecting and reporting | 4 | 4 | 7 (28%) |
| 9 | Support/mentor diverse students | 3 | 3 | 7 (28%) |
| 10 | Work to support a change in mindset for diversity to be at the forefront of all research | 2 | 2 | 7 (28%) |
| 11 | Use the data you already have to investigate samples and empirically demonstrate the power of representative samples, including collaboration on cohort studies | 1 | 1 | 5 (20%) |

***Note.*** Recommendations for individual researcher-level change to improve recruitment and reporting practices in dementia prevention research. Recommendations were generated by Australian dementia researchers and ranked using a multiple criteria decision analysis. ‘Condorcet’ = how many times the recommendation out-ranked all lower-ranked options. ‘Valency’ = how many experts agreed with the position in the rank.
